# Supplementary material for: Attitudes of physicians towards target groups and content of the discharge summary: a cross-sectional analysis in Styria, Austria
Source: BMJ Open. 2019 Dec 17;9(12):e034857. doi: 10.1136/bmjopen-2019-034857 (PMC6937118; doi:10.1136/bmjopen-2019-034857)
Supplement: Author's manuscript [file bmjopen-2019-034857.draft_revisions.pdf]

BMJ Open is committed to open peer review. As part of this commitment we make the peer review history of every article we publish publicly available.

When an article is published we post the peer reviewers' comments and the authors' responses online. We also post the versions of the paper that were used during peer review. These are the versions that the peer review comments apply to.

The versions of the paper that follow are the versions that were submitted during the peer review process. They are not the versions of record or the final published versions. They should not be cited or distributed as the published version of this manuscript.

BMJ Open is an open access journal and the full, final, typeset and author-corrected version of record of the manuscript is available on our site with no access controls, subscription charges or pay-per-view fees (http://bmjopen.bmj.com).

If you have any questions on BMJ Open's open peer review process please email info.bmjopen@bmj.com

BMJ Open

Attitudes of physicians towards target groups and content of the discharge summary– a cross-sectional analysis

Journal:	*BMJ Open*
Manuscript ID	bmjopen-2019-034857
Article Type:	Original research
Date Submitted by the Author:	08-Oct-2019
Complete List of Authors:	Hoffmann, Magdalena; Medical University of Graz, Department of Surgery; Hospital of the Federal State of Styria and University Hospital Graz, Executive Department for Quality and Risk Management<br>Schwarz, Christine; Medical University of Graz, Department of Surgery<br>Pregartner, Gudrun<br>Weinrauch, Maximilian; Medical University of Graz<br>Jantscher, Lydia ; Hospital of the Federal State of Styria and University Hospital Graz, Executive Department for Quality and Risk Management<br>Kamolz, Lars; Medical University of Graz, Department of Surgery<br>Brunner, Gernot; University Hospital Graz, Chief Medical Director<br>Sendlhofer, Gerald; Medical University Graz, Department of Surgery
Keywords:	GENERAL MEDICINE (see Internal Medicine), HEALTH SERVICES ADMINISTRATION & MANAGEMENT, Health & safety < HEALTH SERVICES ADMINISTRATION & MANAGEMENT, Quality in health care < HEALTH SERVICES ADMINISTRATION & MANAGEMENT

SCHOLARONE™
Manuscripts

Attitudes of physicians towards target groups and content of the discharge summary– a cross-sectional analysis

Magdalena Hoffmann[1,2,3], Christine Maria Schwarz[1,2*], Gudrun Pregartner[4], Maximilian Weinrauch[5], Lydia Jantscher[1], Lars-Peter Kamolz[2], Gernot Brunner[2], Gerald Sendlhofer[1,2]

Author Affiliations:

[1]Executive Department for Quality and Risk Management, University Hospital Graz, Austria

[2]Research Unit for Safety in Health, c/o Division of Plastic, Aesthetic and Reconstructive Surgery, Department of Surgery, Medical University of Graz, Austria

[3]Division of Endocrinology and Diabetology, Department of Internal Medicine, Medical University of Graz, Austria

[4]Institute for Medical Informatics, Statistics und Documentation, Medical University of Graz, Austria

[5]Medical University of Graz, Austria

*** Corresponding author:**

Christine Schwarz, MSc. BSc. Research Unit for Safety in Health, c/o Division of Plastic, Aesthetic and Reconstructive Surgery, Department of Surgery, Medical University of Graz, Auenbruggerplatz 1/3, 8036 Graz, Austria, Tel +43664 1188128, Email: Christine.schwarz@medunigraz.at

For peer review only - http://bmjopen.bmj.com/site/about/guidelines.xhtml

First author

Magdalena Hoffmann, MBA, MSc, MSc, PhD magdalena.hoffmann@medunigraz.at

Co-autors

Christine Maria Schwarz, MSc, BSc.: Christine.schwarz@medunigraz.at

Gudrun Pregartner, MSc, BSc : Gudrun.pregartner@medunigraz.at

Maximilian Weinrauch, MD: Maximilian.weinrauch@stud.medunigraz.at

Lydia Jantscher, B.A.: Lydia.jantscher@klinikum-graz.at

Lars-Peter Kamolz, MD, MSc, PhD: Lars.kamolz@medunigraz.at

Gernot Brunner, MD, MSc: Gernot.brunner@medunigraz.at

Gerald Sendlhofer PhD, MSc: Gerald.Sendlhofer@klinikum-graz.at

Short title: Attitudes of physicians concerning the discharge summary

Word count: Abstract (251); Text (2639)

Number of tables: 5

Number of figures: 0

Keywords: Patients, discharge summary, risk, physicians, health literacy

For peer review only - http://bmjopen.bmj.com/site/about/guidelines.xhtml

Strengths and limitations of this study:

- This work investigated the question for whom the discharge summary is to rethink a historical grown attitude.
- A strengths of this study is the total amount of participating physicians (n=1060).
- The discharge summary is primarily considered as a communication tool among physicians; however, patients are also indicated as addressees.
- A limitations of the study is the moderate response rate of external physicians as well as the sample selection, which does not include private/spiritual hospitals or other regions in Austria.

For peer review only

For peer review only - http://bmjopen.bmj.com/site/about/guidelines.xhtml

Abstract

Objectives: The discharge summary represents one of the most important instruments to ensure a safe patient discharge from the hospital. They sometimes have poor quality in content and often include medical jargon, which the patient and their relatives cannot easily understand. Therefore, many risks for patient safety exist. This study investigated the questions for whom the discharge summary is and which contents are necessary to ensure a safe treatment.

Design: cross-sectional analysis

Setting: Styria, Austria

Participants: 3948 internal and external physicians were consulted

Interventions: An online survey consisting of 24 questions was conducted. The survey was distributed to physicians working in the province of Styria, Austria, in 2018 over a period of 6 months.

Main Outcomes and Measures: Attitudes of internal and external physicians in terms of target group, content and health literacy

Results: In total, 1060 physicians participated in the survey. The discharge summary is considered as a communication tool among physicians (97.9%) and the patients are also indicated as addressees (73.5%). Furthermore, there is a high level of agreement that understandable information in the discharge summary leads to fewer questions of the patients (67.9%).

Conclusion: In conclusion, the discharge summary is not only seen as a document for the further treating physician but is also relevant for the patient. Incorporating the patient into their treatment at all levels may possibly strengthen the individual health literacy of the patient and their caring relatives.

For peer review only - http://bmjopen.bmj.com/site/about/guidelines.xhtml

For peer review only

Trial registration: The study was approved by the Medical University Graz Ethics Committee (EK 30-220 ex 17/18).

For peer review only

For peer review only - http://bmjopen.bmj.com/site/about/guidelines.xhtml

Introduction

In order to ensure a safe patient discharge from the hospital in terms of offering all relevant information, the discharge summary (DS) represents one of the most important instruments for this purpose. It usually contains diagnosis, recommendations for further therapy and treatment, monitoring appointments as well as other important information for the therapy of patients. A written DS is legally required and necessary because patients often struggle to accurately recall medical information given by the physician, especially when they are old or anxious. Patients tend to focus on diagnosis-related information and fail to register treatment instructions (1,2). Therefore, the DS should be delivered to the patient and general practitioner (GP) to ensure a continuous treatment of the patient after discharge from hospital (3). In practice, DS are often of poor quality in terms of information content and/or utilise medical jargon such as unexplained abbreviations of medical terms, which the patient and their relatives and even GP struggle to comprehend (4,5). Choudhry *et al.* (2016) reported that for example in Rochester, United States of America, trauma patient DS are written at a too advanced educational level (6). Problematic consequences for the treatment continuity can be manifold if the comprehensive medical language isn‘t adequately understood.

The European Health Literacy Survey report 2012 indicates that in the Austrian population the health literacy is below European Union average (7). In addition, DS in Austria are not governed by detailed national standards for content or structure so far. Therefore, hospitals generated their own format and selected specific contents. The legal structure of the electronic health record (ELGA), which also requires a standardisation of DS by law since 2015, is not yet widespread in Austria. The structure specifications of ELGA are only a rough guide for headings (e.g. diagnosis, outcome measurement, medication, summary) in a given order; however, there are hardly any specific contents offered (8).

For peer review only

Furthermore, the lack of a uniform structure and of important contents in the DS is an international issue as two studies of patients with kidney disease have shown (9,10).

Recent studies reported that patients and their relatives actively want to participate in their care and most patients want to understand their DS (11). It is crucial that patients receive easily comprehensible written medical information as well as therapeutically relevant care information in a patient-directed DS. Lin *et al*. (2014) showed in a prospective randomised controlled trial that a simple patient-directed DS delivered during a brief discussion at discharge significantly improved the patient's understanding of their illness and post-discharge recommendations, which were vice versa limited (12). Patient-directed DS are intended to increase patient understanding as well as to improve the doctor- patient relationship (13,14). A multitude of positive effects, such as increased patient satisfaction (15), increased understanding, as well as a greater patient involvement in their care, were shown (16).

The aim of this study was to investigate attitudes and perceptions among physicians in Styria regarding the current DS in terms of who the addressee of the DS is and what the necessary content is to improve the communication from hospital to the further treating GP or other health care workers in the future. The study was carried out by the University Hospital Graz, Austria, and involved physicians in and outside of the University hospital.

Methods

Ethics statement

The Ethics Committee of the Medical University of Graz approved the study (vote#: 30-220 ex17/18).

Reporting

For peer review only - http://bmjopen.bmj.com/site/about/guidelines.xhtml

The research and reporting methodology followed by "Recruitment process and description of the sample having access to the questionnaire" (17) recommended by equator network.

The survey

This cross-sectional survey was based on a literature review about challenges of the medical DS (4) and contained 24 questions on specific topics (e.g. target group, necessary content, contribution to health literacy, need of change) related to the DS. Each item was scored on a four-point Likert-type scale from 1 – totally agree – to 4 – totally disagree – and provided the additional option of 'not relevant'. After a review process of medical professionals, the survey was pre-tested in 10 individuals (nurses, physicians, and staff from the quality and risk management department) several times. A detailed description of the survey is presented in **TABLE 1.** The online survey was distributed via EvaSys (Electric Paper Evaluationssysteme GmbH, Germany, version 7.1) in 2018 over a period of 6 months.

Study Population

Two groups of physicians, those working in public Styrian hospitals (Styrian Hospitals Limited Liability Company (KAGes)) as well as physicians working outside as a consultant in the province of Styria, were asked to complete the survey. The groups are referred to as "internal" and "external", respectively. The internal group consisted of 2387 physicians and the external group of 1561 physicians. Both groups were invited via Email to the survey. Three reminders within four weeks were sent to non-responders.

Statistical analysis

The survey data were pooled into the categories "agree" (scores 1/2) and "disagree" (scores 3/4) and were descriptively analysed using absolute and relative frequencies. Differences between the two

For peer review only - http://bmjopen.bmj.com/site/about/guidelines.xhtml

physician groups as well as for gender and years of work experience were determined by Fisher's exact test in an exploratory fashion and a p-value of <0.05 was considered significant. All analyses were performed using R version 3.5.3.

Patient and public involvement

This questionnaire was developed from results of preliminary work with focus group discussions with patients, experts and stakeholders however; this present research was done without patient involvement Our preliminary results were presented on a science to public congress for further discussion.

Results

In total, 1060 physicians participated in the survey; 747 internal physicians (response rate 31%) and 313 external physicians (response rate 20%) provided answers. For each of the 24 questions of the survey, a minimum participation rate of 88% was reached, i.e. the respondent answered with one of the four intended answer categories. A detailed description of the participating physicians is presented in **TABLE 2**.

Overall survey results

Although the DS is primarily considered as a communication tool among physicians (97.9%), patients were also indicated as addressees (73.5%). Furthermore, two thirds of the respondents indicated that easily comprehensible information leads to fewer questions from the patients (67.9%). Regarding the necessary content of the DS, diagnosis (100%), therapy (99.7%), recommendations on further treatment (99.6%), control visits and follow-up appointments (98.7%), prescription of medication (98.5%), and behavioural recommendations for the patients (94.4%) receive most agreement from

For peer review only - http://bmjopen.bmj.com/site/about/guidelines.xhtml

the respondents. Only less than a quarter of participants (22.5%) wish to have specific abbreviations in their DS.

The DS in its current form is felt to contribute only mediocrely to health literacy (49.4%), and most respondents are of the opinion that changes should be made (46.2%). A detailed description of the physicians' ratings is presented in **TABLE 3**.

Differences between internal and external physicians

There were significant differences given by internal and external physicians for seven of the 24 items. Patients were more commonly considered as addressees of the DS by the internal group (75.8% versus 67.8%, p=0.009). The internal group was less frequently of the opinion that a DS that is comprehensible for patients leads to less time-consuming questions (65.8% versus 72.7%, p=0.034) and that patients need not understand the DS (23.9% versus 32.3%, p=0.006). Additionally, less internal physicians received a training in how a DS should be structured (46.8% versus 55.5%, p=0.012). Internal physicians were more often of the opinion that the content of the DS in its current form is sufficient for further treatment (81.0% versus 71.5%, p=0.001) and that the DS contributes to increase the individual health literacy of patients (52.8% versus 41.2%, p=0.001). Whereas less than half of the internal group feels the need to make changes to the current DS, more than half of the external group would like to see changes made (41.4% versus 57.9%, p<0.001). A detailed description of the physicians' ratings is presented in **TABLE 3.**

Differences according to gender

For peer review only - http://bmjopen.bmj.com/site/about/guidelines.xhtml

Four-hundred sixty (43.4%) of the respondents were female, 565 (53.3%) were male, and 35 (3.3%) did not disclose their gender. Female physicians more often see patients (82.2% versus 67.0%, p<0.001) and all persons authorized by the patient (70.4% versus 60.9%, p=0.002) as addressees of the DS. More of them than their male colleagues also believe that a DS that is understandable for patients leads to less time-consuming questions (71.7% versus 65.0%, p=0.029). Conversely, less female physicians agree with that the patient need not understand the DS (18.9% versus 31.8%, p<0.001) or that abbreviations are a necessary part of the DS (17.2% versus 26.0%, p=0.001). Interestingly, more female physicians want therapy recommendations or reports from other health care professionals, such as clinical social workers (59.5% versus 51.1%, p=0.010) or medical-technical services (72.0% versus 65.0%, p=0.022), to be included in the DS. A detailed description of the responses by gender is presented in **TABLE 4.**

Differences according to years of work experience

Three-hundred nine (29.2%) of the respondents had a work experience of up to ten years, 274 (25.8%) had been working for 11 to 20 years, and 461 (43.5%) for more than 20 years; only 16 (1.5%) did not disclose this information. Physicians with up to 20 years of work experience see the patient more often as addressee of the DS than those who have been working in the field longer (80.5% of physicians with a work experience of 0-10 years versus 79.3% of those working 11-20 years versus 64% of those working >20 years, p<0.001). Physicians with less professional experience also tend to agree less with the statement that the DS does not need to be understood by patients (16.7% versus 23.5% versus 34.6%, p<0.001). The groups also showed different rates of agreement to the statement that, in future, the DS should contain further procedures and treatment goals in an understandable way, so that patients themselves can contribute to the improvement of their health (81.1% versus

For peer review only

For peer review only - http://bmjopen.bmj.com/site/about/guidelines.xhtml

84.2% versus 75.2%, p=0.011). Physicians with more work experience were more open to changes of the DS than younger ones (39.3% versus 49.0% versus 49.6%, p=0.016). A detailed description of the responses by work experience is presented in **TABLE 5.**

Discussion

The main finding of this cross-sectional survey is that physicians consider themselves as the target group of the DS. The physicians have a clear view regarding the required content in the DS. However, gender and years of work experience also influence the results.

Implications of findings

This survey showed that most physicians consider themselves as the target group of the DS; however, patients, who according to the Austrian law are the owners of the written DS (18), are also often indicated as a target group. Patient-comprehensible DS support patients and relatives in understanding medical information as they include the most important messages when leaving the hospital. A very early study by Shankar in 2003 stated that simple and specific instructions for patients are better recalled than general statements. Patient comprehension can be aided with written or visual material (1). Health literacy means finding, understanding, and using health information (19). However, fewer than half of physicians said that the current DS supports health literacy of patients. A patient-directed DS could support finding the right information and understanding the offered health information (11,20,21). Positive effects of a patient-directed DS were reported by Lin *et al.* in 2014. Patients who received a patient-directed DS had an immediate increase in the understanding of tests performed at the hospital as well as a higher compliance in post-discharge recommendations (11). Wernick *et al.* reported in 2016 that minimising the use of medical

For peer review only - http://bmjopen.bmj.com/site/about/guidelines.xhtml

For peer review only

terminology in medical correspondence significantly improved patient understanding and perception of their ability to manage chronic health conditions (16). Our results also showed that the majority of physicians consider medical terminology a necessary part of DS but disapprove the use of abbreviations.

Our results show that physicians have a clear expectation regarding the required content in the DS. The need for specific content in Styria is similar to other studies (22). The use of ELGA criteria, providing a minimum number of (mandatory and optional) headings in a fixed structure, to guide physicians in writing a comprehensive DS holds promise in improving the existing deficits in communication at transitions of care, as another study using a standardized DS already showed (14).

For a majority of physicians, abbreviations are undesirable since they are not only problematic for patients but also for physicians. Chemali *et al.* reported in 2015 that abbreviations used in hospital DS are not well understood by the GPs who receive them (17). In another study of Shilo *et al.* abbreviations from 80 DS and admission notes from orthopaedic surgery and medical wards were extracted and graded by senior physicians as understandable, ambiguous, or unknown (23). To improve comprehension of the DS the use of (ambiguous and unknown) abbreviations should be decreased.

It is noteworthy that female physicians expressed that the DS should contain more reports from other health care workers. Furthermore, they see a greater need for patients to understand the DS than male physicians do. The differences in answers related to the years of work experience may indicate a paradigm shift. Physicians with fewer years of experience see the patient significantly more often as addressee of the DS compared to physicians with more than 20 years of work experience. The authors are not aware of comparable findings from current literature.

For peer review only - http://bmjopen.bmj.com/site/about/guidelines.xhtml

Our results showed that less than half of the physicians had received training to write the DS. However, physicians with more years of work experience more frequently stated to have received specific training as part of their education. In a study by Yemm *et al*. in 2014, 36% of young physicians reported that they had inadequate training in composing a DS (15). Therefore, it may be an advantage for young medical professionals to have a fixed structure and guidelines for its content. However, a good structure and layout does not replace the training in writing medical DS. A study by Shivji *et al*. has demonstrated that educational sessions improved the quality of written DS (24). We suggest that a course in writing DS should be included as part of the curriculum in medical schools, as it is important for junior doctors to learn how to prepare a DS.

Furthermore, to improve the DS, supporting instruments and standards need to be implemented. In 2018 Savvopoulos *et al*. developed a scoring tool to assess the quality of the DS (16). Such tools can help to assess and improve the quality of current DS and derive any need for improvement if necessary.

Strengths and limitations of the study

A strength of this study is the high response rate of internal physicians and the involvement of occupational groups and patients in the project process before and after the survey.

This study has several limitations. This survey is a snapshot of current attitudes of doctors regarding discharge information. Another limitation are the dropped non-responders of the survey. A limitation of the present study is the sample selection, which does not include private/spiritual hospitals or other regions in Austria. Another important limitation of our study is the moderate response rate of external

For peer review only - http://bmjopen.bmj.com/site/about/guidelines.xhtml

physicians. However, this phenomenon was also observed in other surveys with externals. It might be that external physicians can hardly be attracted for participation.

Conclusions

Based on our results, it can be concluded that the DS is perceived not only as a document for the further treating physician but also for the patient and other caregivers. Now, the discharge summary primarily is written for the further treating doctor and has often not a uniform structure. The question arises how a discharge summary can be designed, that is partly understandable for the patient and relatives and which can be implemented in the clinic practice. Further research on this topic should address this question. An international comparison of physicians' perceptions, attitudes, and solutions regarding a patient-directed DS, important contents, and improvements in health literacy of patients would be beneficial.

For peer review only

For peer review only - http://bmjopen.bmj.com/site/about/guidelines.xhtml

References

1. Shankar J. Patients' memory for medical information [3]. J R Soc Med. 2003;96(10):520.

2. Kessels RPC. Patients' memory for medical information. J R Soc Med [Internet]. 2003 May [cited 2019 Jul 26];96(5):219–22. Available from: http://www.ncbi.nlm.nih.gov/pubmed/12724430

3. BMASGK. Zielsteuerung-Gesundheit Qualitätsstandard Aufnahme-und Entlassungs-management [Internet]. [cited 2019 May 24]. Available from: https://www.sozialministerium.at/cms/site/attachments/1/3/0/CH3970/CMS1350910195632/qualitaetsstandard_aufem.pdf

4. Schwarz CM, Hoffmann M, Schwarz P, Kamolz L, Brunner G, Sendlhofer G. A systematic literature review and narrative synthesis on the risks of medical discharge letters for patients ' safety. 2019;1:1–10.

5. Sarzynski E, Hashmi H, Subramanian J, Fitzpatrick L, Polverento M, Simmons M, et al. Opportunities to improve clinical summaries for patients at hospital discharge. BMJ Qual Saf [Internet]. 2016 May 6 [cited 2019 Jul 26];26(5):bmjqs-2015-005201. Available from: http://www.ncbi.nlm.nih.gov/pubmed/27154878

6. Choudhry AJ, Baghdadi YMK, Wagie AE, Habermann EB, Heller SF, Jenkins DH, et al. Readability of discharge summaries: With what level of information are we dismissing our patients? In: American Journal of Surgery. 2016. p. 631–6.

7. Sorensen K, Pelikan JM, Rothlin F, Ganahl K, Slonska Z, Doyle G, et al. Health literacy in Europe: comparative results of the European health literacy survey (HLS-EU). Eur J Public

For peer review only - http://bmjopen.bmj.com/site/about/guidelines.xhtml

Health. 2015;25(6):1–6.

8. Sabutsch S, Brandstätter J. ELGA CDA Implementierungsleitfäden. 2013;1–216. Available from: https://www.gesundheit.gv.at/Portal.Node/ghp/public/content/ELGA/elga-was-ist-elga.html

9. Harel Z, Wald R, Perl J, Schwartz D, Bell CM. Evaluation of deficiencies in current discharge summaries for dialysis patients in Canada. J Multidiscip Healthc. 2012;5:77–84.

10. Greer RC, Liu Y, Crews DC, Jaar BG, Rabb H, Boulware LE. Hospital discharge communications during care transitions for patients with acute kidney injury: a cross-sectional study. BMC Health Serv Res [Internet]. 2016 Dec 30 [cited 2019 Aug 1];16(1):449. Available from: http://www.ncbi.nlm.nih.gov/pubmed/27577888

11. Hammerer P. Patientenverständliche Arztbriefe und Befunde. Springer Medizin. 2018;

12. Lin R, Gallagher R, Spinaze M, Najoumian H, Dennis C, Clifton-Bligh R, et al. Effect of a patient-directed discharge letter on patient understanding of their hospitalisation. Intern Med J [Internet]. 2014 Sep 1 [cited 2018 Jan 29];44(9):851–7. Available from: http://doi.wiley.com/10.1111/imj.12482

13. White P, Singleton A, Jones R. Copying referral letters to patients: The views of patients, patient representatives and doctors. Patient Educ Couns. 2004;55(1):94–8.

14. Minhas R. Does copying clinical or sharing correspondence to patients result in better care? Vol. 61, International Journal of Clinical Practice. 2007. p. 1390–5.

15. O'Driscoll BR, Koch J, Paschalides C. Copying letters to patients: most patients want copies of letters from outpatient clinics and find them useful. Vol. 327, BMJ (Clinical research ed.).

For peer review only

For peer review only - http://bmjopen.bmj.com/site/about/guidelines.xhtml

2003. p. 451.

16. VanSuch M, Naessens JM, Stroebel RJ, Huddleston JM, Williams AR. Effect of discharge instructions on readmission of hospitalised patients with heart failure: Do all of the Joint Commission on Accreditation of Healthcare Organizations heart failure core measures reflect better care? Qual Saf Heal Care. 2006;15(6):414–7.

17. Kelley K, Clark B, Brown V, Sitzia J. Recruitment process and description of the sample having access to the questionnaire. [cited 2019 Sep 25]; Available from: https://watermark.silverchair.com/mzg031.pdf?token=AQECAHi208BE49Ooan9kkhW_Ercy7Dm3ZL_9Cf3qfKAc485ysgAAAmAwggJcBgkqhkiG9w0BBwagggJNMIICSQIBADCCAkIGCSqGSIb3DQEHATAeBglghkgBZQMEAS4wEQQMvJGQAvtFfKg1-e_MAgEQgIICE6gwobbp5J3ssq2wiiOTQIciDtT5-Ep5xxNYnBo9koEgaxdb

18. Kuranstaltengesetz Kundmachungsorgan K. Krankenanstalten- u. Kuranstaltengesetz § 24 [Internet]. Vol. 124. 2009 [cited 2018 Jan 30]. Available from: www.ris.bka.gv.at

19. Sørensen K, Van Den Broucke S, Fullam J, Doyle G, Pelikan J. Health literacy and public health: A systematic review and integration of definitions and models. BMC Public Health [Internet]. 2012 [cited 2018 Mar 26];12:80. Available from: https://bmcpublichealth.biomedcentral.com/track/pdf/10.1186/1471-2458-12-80?site=bmcpublichealth.biomedcentral.com

20. Bench SD, Heelas K, White C, Griffiths P. Providing critical care patients with a personalised discharge summary: A questionnaire survey and retrospective analysis exploring feasibility and effectiveness. Intensive Crit Care Nurs. 2014;30(2):69–76.

21. Buurman BM, Verhaegh KJ, Smeulers M, Vermeulen H, Geerlings SE, Smorenburg S, et al.

For peer review only

Improving handoff communication from hospital to home: the development, implementation and evaluation of a personalized patient discharge letter. Int J Qual Heal Care [Internet]. 2016 Jun [cited 2018 Jun 7];28(3):384–90. Available from: http://www.ncbi.nlm.nih.gov/pubmed/27224995

22. Sorita A, Robelia PM, Kattel SB, McCoy CP, Keller AS, Almasri J, et al. The Ideal Hospital Discharge Summary. J Patient Saf. 2017;1.

23. Shilo L, Shilo G. Analysis of abbreviations used by residents in admission notes and discharge summaries. QJM. 2018;111(3):179–83.

24. Shivji FS, Ramoutar DN, Bailey C, Hunter JB. Improving communication with primary care to ensure patient safety post-hospital discharge. Br J Hosp Med. 2015;76(1):46–9.

For peer review only

For peer review only - http://bmjopen.bmj.com/site/about/guidelines.xhtml

TABLES

TABLE 1: Content of the survey

Item	*Questions*
2	**The DS is a communication tool …**
2.1	... among physicians.
2.2	… for the information of patients.
2.3	... for all persons authorized by the patient (legal persons, relatives, caregivers).
2.4	The DS that is comprehensible for patients leads to less time-consuming questions.
2.5	The DS is usually not read by patients.
2.6	The patient needn´t understand the DS, as it is explained by the further treating physicians.
2.7	As part of my education I have attended relevant training courses in the field of communication, such as dealing with patients and relatives.
2.8	In my education as a physician, the structure and content of the DS was an integral part.
2.9	I regularly attend training courses on communication.
3	**The following are necessary contents in the DS …**
3.1	... diagnosis
3.2	... therapy
3.3	… medical terminology
3.4	... specific abbreviations
3.5	... recommendations on further treatment
3.6	... prescription of medication
3.7	... control visits and follow-up appointments
3.8	... behavioral recommendations for the patients
3.9	…therapy recommendations / report from graduated health and nursing staff
3.10	…therapy recommendations / report from the clinical social workers
3.11	…therapy recommendations / report from the medical-technical services (e.g. dietology, physiotherapy)
3.12	The content of the DS in its current form is sufficient for further treatment.
3.13	The DS in its present form contributes to increase the individual health literacy of patients.
4.1	In future, the DS should contain further procedures and treatment goals in an understandable way, so that patients themselves can contribute to the improvement of their health.
4.2	Should the DS be changed in its current form?

For peer review only

374 **TABLE 2: Baseline characteristics of the participating physicians, presented separately for**

375 **internal and external physicians**

Variable	*Category*	*Internal (n=747)*	*External (n=313)*	*Total (n=1060)*
Gender	Female	346 (46.3%)	114 (36.4%)	460 (43.4%)
	Male	371 (49.7%)	194 (62%)	565 (53.3%)
	(Missing)	30 (4%)	5 (1.6%)	35 (3.3%)
Work experience in years	0-10	276 (36.9%)	33 (10.5%)	309 (29.2%)
	11-20	201 (26.9%)	73 (23.3%)	274 (25.8%)
	>20	258 (34.5%)	203 (64.9%)	461 (43.5%)
	(Missing)	12 (1.6%)	4 (1.3%)	16 (1.5%)
Level of work experience	Junior physicians	37 (5%)	-	37 (3.5%)
	Ward physicians	163 (21.8%)	-	163 (15.4%)
	Assistant physicians	349 (46.7%)	-	349 (32.9%)
	Senior physicians	176 (23.6%)	-	176 (16.6%)
	(Missing)	22 (2.9%)	313 (100%)	335 (31.6%)
Field of work	General practitioner	-	138 (44.1%)	138 (13%)
	Specialist	-	152 (48.6%)	152 (14.3%)
	(Missing)	747 (100%)	23 (7.3%)	770 (72.6%)

376 Percentages pertain to available responses.

377

378

379

380

381

382

383

384

385

386

387

388

389

390

391

392

For peer review only

For peer review only - http://bmjopen.bmj.com/site/about/guidelines.xhtml

393 **TABLE 3. Responses to the survey items, presented separately for internal and external**

394 **physicians**

395

396

For peer review only

For peer review only - http://bmjopen.bmj.com/site/about/guidelines.xhtml

Item	*Category*	*Internal*	*External*	*Total*	*p-value*
2. **The DS is a communication tool …**					
2.1 ... among physicians.	1/2	728 (98.1%)	303 (97.4%)	1031 (97.9%)	0.483
	3/4	14 (1.9%)	8 (2.6%)	22 (2.1%)	
2.2 … for the information of patients.	1/2	559 (75.8%)	208 (67.8%)	767 (73.5%)	0.009*
	3/4	178 (24.2%)	99 (32.2%)	277 (26.5%)	
2.3 ... for all persons authorized by the patient (legal persons, relatives, caregivers).	1/2	478 (66.5%)	186 (61.4%)	664 (65%)	0.132
	3/4	241 (33.5%)	117 (38.6%)	358 (35%)	
2.4 The DS that is comprehensible for patients leads to less time-consuming questions.	1/2	467 (65.8%)	224 (72.7%)	691 (67.9%)	0.034*
	3/4	243 (34.2%)	84 (27.3%)	327 (32.1%)	
2.5 The DS is usually not read by patients.	1/2	315 (47.2%)	145 (48.3%)	460 (47.5%)	0.781
	3/4	353 (52.8%)	155 (51.7%)	508 (52.5%)	
2.6 The patient needn´t understand the DS, as it is explained by the further treating physicians.	1/2	173 (23.9%)	100 (32.3%)	273 (26.4%)	0.006*
	3/4	551 (76.1%)	210 (67.7%)	761 (73.6%)	
2.7 As part of my education I have attended relevant training courses in the field of communication, such as dealing with patients and relatives.	1/2	347 (48.1%)	147 (49.7%)	494 (48.5%)	0.679
	3/4	375 (51.9%)	149 (50.3%)	524 (51.5%)	
2.8 In my education as a physician, the structure and content of the DS was an integral part	1/2	341 (46.8%)	171 (55.5%)	512 (49.4%)	0.012*
	3/4	388 (53.2%)	137 (44.5%)	525 (50.6%)	
2.9 I regularly attend training courses on communication.	1/2	227 (31%)	106 (34.6%)	333 (32.1%)	0.274
	3/4	506 (69%)	200 (65.4%)	706 (67.9%)	
3. **The following are necessary contents in the DS …**					
3.1 ... diagnosis	1/2	733 (100%)	311 (100%)	1044 (100%)	1.000
	3/4	0 (0%)	0 (0%)	0 (0%)	
3.2 ... therapy	1/2	730 (99.6%)	310 (100%)	1040 (99.7%)	0.559
	3/4	3 (0.4%)	0 (0%)	3 (0.3%)	
3.3 … medical terminology	1/2	493 (68.7%)	228 (74.5%)	721 (70.4%)	0.062
	3/4	225 (31.3%)	78 (25.5%)	303 (29.6%)	
3.4 ... specific abbreviations	1/2	162 (22.8%)	65 (21.7%)	227 (22.5%)	0.742
	3/4	547 (77.2%)	235 (78.3%)	782 (77.5%)	
3.5 ... recommendations on further treatment	1/2	729 (99.6%)	308 (99.7%)	1037 (99.6%)	1.000
	3/4	3 (0.4%)	1 (0.3%)	4 (0.4%)	
3.6 ... prescription of medication	1/2	720 (98.5%)	303 (98.4%)	1023 (98.5%)	1.000
	3/4	11 (1.5%)	5 (1.6%)	16 (1.5%)	
3.7 ... control visits and follow-up appointments	1/2	721 (99%)	303 (97.7%)	1024 (98.7%)	0.137
	3/4	7 (1%)	7 (2.3%)	14 (1.3%)	
3.8 ... behavioral recommendations for the patients	1/2	686 (95.3%)	282 (92.5%)	968 (94.4%)	0.075
	3/4	34 (4.7%)	23 (7.5%)	57 (5.6%)	
3.9 …therapy recommendations / report from graduated health and nursing staff	1/2	326 (46.7%)	160 (52.8%)	486 (48.6%)	0.085

For peer review only

	3/4	372 (53.3%)	143 (47.2%)	515 (51.4%)	
3.10 …therapy recommendations / report from the clinical social workers	1/2	375 (53.6%)	171 (56.8%)	546 (54.5%)	0.368
	3/4	325 (46.4%)	130 (43.2%)	455 (45.5%)	
3.11 …therapy recommendations / report from the medical-technical services (e.g. dietology, physiotherapy)	1/2	473 (67.3%)	207 (69.2%)	680 (67.9%)	0.555
	3/4	230 (32.7%)	92 (30.8%)	322 (32.1%)	
3.12 The content of the DS in its current form is sufficient for further treatment.	1/2	571 (81%)	216 (71.5%)	787 (78.2%)	0.001*
	3/4	134 (19%)	86 (28.5%)	220 (21.8%)	
3.13 The DS in its present form contributes to increase the individual health literacy of patients.	1/2	346 (52.8%)	115 (41.2%)	461 (49.4%)	0.001*
	3/4	309 (47.2%)	164 (58.8%)	473 (50.6%)	
4.1. In future, the DS should contain further procedures and treatment goals in an understandable way, so that patients themselves can contribute to the improvement of their health.	1/2	573 (79.6%)	240 (78.7%)	813 (79.3%)	0.737
	3/4	147 (20.4%)	65 (21.3%)	212 (20.7%)	
4.2 Should the DS be changed in its current form?	1/2	284 (41.4%)	165 (57.9%)	449 (46.2%)	<0.001*
	3/4	402 (58.6%)	120 (42.1%)	522 (53.8%)	

Legend: * significant values, missing data can occur because of nonresponse and is not explicitly stated; percentages pertain to available responses.

For peer review only

TABLE 4. Responses to the survey items according to gender

Item	*Category*	*Female*	*Male*	*Total*	*p-value*
2. **The DS is a communication tool …**					
2.1 ... among physicians.	1/2	451 (98%)	548 (97.7%)	1031 (97.9%)	0.829
	3/4	9 (2%)	13 (2.3%)	22 (2.1%)	
2.2 … for the information of patients.	1/2	374 (82.2%)	373 (67%)	767 (73.5%)	<0.001*
	3/4	81 (17.8%)	184 (33%)	277 (26.5%)	
2.3 ... for all persons authorized by the patient (legal persons, relatives, caregivers).	1/2	309 (70.4%)	336 (60.9%)	664 (65%)	0.002*
	3/4	130 (29.6%)	216 (39.1%)	358 (35%)	
2.4 The DS that is comprehensible for patients leads to less time-consuming questions.	1/2	321 (71.7%)	353 (65%)	691 (67.9%)	0.029*
	3/4	127 (28.3%)	190 (35%)	327 (32.1%)	
2.5 The DS is usually not read by patients.	1/2	194 (46.3%)	254 (48.8%)	460 (47.5%)	0.470
	3/4	225 (53.7%)	267 (51.2%)	508 (52.5%)	
2.6 The patient needn´t understand the DS, as it is explained by the further treating physicians.	1/2	86 (18.9%)	174 (31.8%)	273 (26.4%)	<0.001*
	3/4	368 (81.1%)	374 (68.2%)	761 (73.6%)	
2.7 As part of my education I have attended relevant training courses in the field of communication, such as dealing with patients and relatives.	1/2	207 (46.8%)	273 (50.1%)	494 (48.5%)	0.337
	3/4	235 (53.2%)	272 (49.9%)	524 (51.5%)	
2.8 In my education as a physician, the structure and content of the DS was an integral part	1/2	209 (46.4%)	286 (51.5%)	512 (49.4%)	0.113
	3/4	241 (53.6%)	269 (48.5%)	525 (50.6%)	
2.9 I regularly attend training courses on communication.	1/2	142 (31.1%)	179 (32.5%)	333 (32.1%)	0.684
	3/4	314 (68.9%)	372 (67.5%)	706 (67.9%)	
3. **The following are necessary contents in the DS …**					
3.1 ... diagnosis	1/2	455 (100%)	557 (100%)	1044 (100%)	1.000
	3/4	0 (0%)	0 (0%)	0 (0%)	
3.2 ... therapy	1/2	453 (99.8%)	555 (99.6%)	1040 (99.7%)	1.000
	3/4	1 (0.2%)	2 (0.4%)	3 (0.3%)	
3.3 … medical terminology	1/2	310 (69.5%)	387 (70.7%)	721 (70.4%)	0.676
	3/4	136 (30.5%)	160 (29.3%)	303 (29.6%)	
3.4 ... specific abbreviations	1/2	76 (17.2%)	140 (26%)	227 (22.5%)	0.001*
	3/4	365 (82.8%)	398 (74%)	782 (77.5%)	
3.5 ... recommendations on further treatment	1/2	450 (99.6%)	555 (99.6%)	1037 (99.6%)	1.000
	3/4	2 (0.4%)	2 (0.4%)	4 (0.4%)	
3.6 ... prescription of medication	1/2	448 (98.7%)	543 (98.2%)	1023 (98.5%)	0.619

For peer review only

	3/4	6 (1.3%)	10 (1.8%)	16 (1.5%)	
3.7 ... control visits and follow-up appointments	1/2	447 (98.7%)	547 (98.7%)	1024 (98.7%)	1.000
	3/4	6 (1.3%)	7 (1.3%)	14 (1.3%)	
3.8 ... behavioral recommendations for the patients	1/2	420 (94.2%)	517 (94.5%)	968 (94.4%)	0.890
	3/4	26 (5.8%)	30 (5.5%)	57 (5.6%)	
3.9 …therapy recommendations / report from graduated health and nursing staff	1/2	218 (50.5%)	252 (46.7%)	486 (48.6%)	0.246
	3/4	214 (49.5%)	288 (53.3%)	515 (51.4%)	
3.10 …therapy recommendations / report from the clinical social workers	1/2	260 (59.5%)	274 (51.1%)	546 (54.5%)	0.010*
	3/4	177 (40.5%)	262 (48.9%)	455 (45.5%)	
3.11 …therapy recommendations / report from the medical-technical services (e.g. dietology, physiotherapy)	1/2	314 (72%)	349 (65%)	680 (67.9%)	0.022*
	3/4	122 (28%)	188 (35%)	322 (32.1%)	
3.12 The content of the DS in its current form is sufficient for further treatment.	1/2	333 (76.2%)	431 (80%)	787 (78.2%)	0.161
	3/4	104 (23.8%)	108 (20%)	220 (21.8%)	
3.13 The DS in its present form contributes to increase the individual health literacy of patients.	1/2	192 (47.9%)	252 (49.9%)	461 (49.4%)	0.548
	3/4	209 (52.1%)	253 (50.1%)	473 (50.6%)	
4.1. In future, the DS should contain further procedures and treatment goals in an understandable way, so that patients themselves can contribute to the improvement of their health.	1/2	364 (81.6%)	426 (77.9%)	813 (79.3%)	0.155
	3/4	82 (18.4%)	121 (22.1%)	212 (20.7%)	
4.2 Should the DS be changed in its current form?	1/2	198 (46%)	238 (46.3%)	449 (46.2%)	0.948
	3/4	232 (54%)	276 (53.7%)	522 (53.8%)	

Legend: * significant values, missing data can occur because of nonresponse and is not explicitly stated; percentages pertain to available responses.

For peer review only

TABLE 5. Responses to the survey items according to years of work experience

Item	*Category*	*0-10*	*11-20*	*>20*	*Total*	*p-value*
**2. The DS is a communication tool …**						
2.1 ... among physicians.	1/2	303 (98.4%)	261 (96%)	454 (98.7%)	1031 (97.9%)	0.050
	3/4	5 (1.6%)	11 (4%)	6 (1.3%)	22 (2.1%)	
2.2 … for the information of patients.	1/2	248 (80.5%)	214 (79.3%)	293 (64.8%)	767 (73.5%)	<0.001*
	3/4	60 (19.5%)	56 (20.7%)	159 (35.2%)	277 (26.5%)	
2.3 ... for all persons authorized by the patient (legal persons, relatives, caregivers).	1/2	201 (68.4%)	188 (69.4%)	264 (59.5%)	664 (65%)	0.008
	3/4	93 (31.6%)	83 (30.6%)	180 (40.5%)	358 (35%)	
2.4 The DS that is comprehensible for patients leads to less time-consuming questions.	1/2	207 (69.2%)	193 (73.1%)	281 (63.6%)	691 (67.9%)	0.026*
	3/4	92 (30.8%)	71 (26.9%)	161 (36.4%)	327 (32.1%)	
2.5 The DS is usually not read by patients.	1/2	157 (56.3%)	119 (47.6%)	178 (41.9%)	460 (47.5%)	0.001*
	3/4	122 (43.7%)	131 (52.4%)	247 (58.1%)	508 (52.5%)	
2.6 The patient needn´t understand the DS, as it is explained by the further treating physicians.	1/2	51 (16.7%)	62 (23.5%)	156 (34.6%)	273 (26.4%)	<0.001*
	3/4	254 (83.3%)	202 (76.5%)	295 (65.4%)	761 (73.6%)	
2.7 As part of my education I have attended relevant training courses in the field of communication, such as dealing with patients and relatives.	1/2	151 (50.3%)	115 (43.7%)	222 (50.1%)	494 (48.5%)	0.196
	3/4	149 (49.7%)	148 (56.3%)	221 (49.9%)	524 (51.5%)	
2.8 In my education as a physician, the structure and content of the DS was an integral part	1/2	128 (42.5%)	137 (50.7%)	241 (53.2%)	512 (49.4%)	0.014*
	3/4	173 (57.5%)	133 (49.3%)	212 (46.8%)	525 (50.6%)	
2.9 I regularly attend training courses on communication.	1/2	74 (24.3%)	88 (32.8%)	167 (36.9%)	333 (32.1%)	0.001*
	3/4	230 (75.7%)	180 (67.2%)	286 (63.1%)	706 (67.9%)	
**3. The following are necessary contents in the DS …**						
3.1 ... diagnosis	1/2	304 (100%)	273 (100%)	454 (100%)	1044 (100%)	1.000
	3/4	0 (0%)	0 (0%)	0 (0%)	0 (0%)	
3.2 ... therapy	1/2	303 (99.7%)	271 (100%)	453 (99.6%)	1040 (99.7%)	0.794
	3/4	1 (0.3%)	0 (0%)	2 (0.4%)	3 (0.3%)	
3.3 … medical terminology	1/2	196 (64.9%)	194 (72.4%)	321 (72.6%)	721 (70.4%)	0.055
	3/4	106 (35.1%)	74 (27.6%)	121 (27.4%)	303 (29.6%)	
3.4 ... specific abbreviations	1/2	58 (19.6%)	64 (24.1%)	103 (23.6%)	227 (22.5%)	0.347
	3/4	238 (80.4%)	202 (75.9%)	334 (76.4%)	782 (77.5%)	
3.5 ... recommendations on further treatment	1/2	303 (99.7%)	269 (99.3%)	453 (99.8%)	1037 (99.6%)	0.696
	3/4	1 (0.3%)	2 (0.7%)	1 (0.2%)	4 (0.4%)	
3.6 ... prescription of medication	1/2	300 (99%)	266 (98.2%)	444 (98.2%)	1023 (98.5%)	0.655

For peer review only - http://bmjopen.bmj.com/site/about/guidelines.xhtml

	3/4	3 (1%)	5 (1.8%)	8 (1.8%)	16 (1.5%)	
3.7 ... control visits and follow-up appointments	1/2	299 (99.7%)	270 (99.3%)	442 (97.6%)	1024 (98.7%)	0.041*
	3/4	1 (0.3%)	2 (0.7%)	11 (2.4%)	14 (1.3%)	
3.8 ... behavioral recommendations for the patients	1/2	290 (97.3%)	254 (94.1%)	411 (92.6%)	968 (94.4%)	0.016*
	3/4	8 (2.7%)	16 (5.9%)	33 (7.4%)	57 (5.6%)	
3.9 …therapy recommendations / report from graduated health and nursing staff	1/2	133 (45.2%)	124 (48.1%)	221 (50.6%)	486 (48.6%)	0.364
	3/4	161 (54.8%)	134 (51.9%)	216 (49.4%)	515 (51.4%)	
3.10 …therapy recommendations / report from the clinical social workers	1/2	166 (56.3%)	134 (51.3%)	239 (55.1%)	546 (54.5%)	0.476
	3/4	129 (43.7%)	127 (48.7%)	195 (44.9%)	455 (45.5%)	
3.11 …therapy recommendations / report from the medical-technical services (e.g. dietology, physiotherapy)	1/2	201 (67.9%)	168 (65.1%)	302 (69.1%)	680 (67.9%)	0.552
	3/4	95 (32.1%)	90 (34.9%)	135 (30.9%)	322 (32.1%)	
3.12 The content of the DS in its current form is sufficient for further treatment.	1/2	234 (79.6%)	203 (78.1%)	340 (77.1%)	787 (78.2%)	0.724
	3/4	60 (20.4%)	57 (21.9%)	101 (22.9%)	220 (21.8%)	
3.13 The DS in its present form contributes to increase the individual health literacy of patients.	1/2	145 (52.3%)	129 (52.7%)	182 (45.3%)	461 (49.4%)	0.095
	3/4	132 (47.7%)	116 (47.3%)	220 (54.7%)	473 (50.6%)	
4.1. In future, the DS should contain further procedures and treatment goals in an understandable way, so that patients themselves can contribute to the improvement of their health.	1/2	245 (81.1%)	224 (84.2%)	333 (75.2%)	813 (79.3%)	0.011*
	3/4	57 (18.9%)	42 (15.8%)	110 (24.8%)	212 (20.7%)	
4.2 Should the DS be changed in its current form?	1/2	114 (39.3%)	121 (49%)	209 (49.6%)	449 (46.2%)	0.016*
	3/4	176 (60.7%)	126 (51%)	212 (50.4%)	522 (53.8%)	

Legend: * significant values, missing data can occur because of nonresponse and is not explicitly stated; percentages pertain to available responses.

For peer review only - http://bmjopen.bmj.com/site/about/guidelines.xhtml

Declarations

Contributors:

Gurantor: MH

Study concept and design: MH, GB, MW

Acquisition of data: MH, CS, GP, LJ, LK

Analysis and interpretation of pooled data: MH, CS, GP, LJ, LK

Drafting of the manuscript: MH, CS

Critical revision of the manuscript for important intellectual content: All authors

Statistical analysis of pooled data: GP, LJ

Funding: This work was supported by the Healthcare fund Styria (Gesundheitsfonds Steiermark). The funders had no role in the study design, data collection, data analysis, data interpretation, or writing of the report.

Competing interests: All authors have completed the ICMJE uniform disclosure form at http://icmje.org/conflicts-of-interest/and declared no conflict of interests.

Patient consent for publication: Not required.

Ethics approval: The Ethics Committee of the Medical University of Graz approved the study (vote#: 30-220 ex17/18).

Transparency declaration: The lead author (MH) affirms that this manuscript is an honest, accurate, and transparent account of the study being reported; that no important aspects of the study have been omitted; and that any discrepancies from the study as planned have been explained.

For peer review only

For peer review only - http://bmjopen.bmj.com/site/about/guidelines.xhtml

432 **Consent for publication**: All authors confirmed the publication in its present form.

433 **Data availability statement:** The datasets used and/or analyzed during the current study are
434 available from the corresponding author on reasonable request.

For peer review only

For peer review only - http://bmjopen.bmj.com/site/about/guidelines.xhtml

International Journal for Quality in Health Care 2003; Volume 15, Number 3: pp. 261–266 10.1093/intqhc/mzg031

Methodology Matters

Good practice in the conduct and reporting of survey research

KATE KELLEY, BELINDA CLARK, VIVIENNE BROWN AND JOHN SITZIA

Research Department, Worthing & Southlands Hospitals NHS Trust, Worthing, West Sussex, UK

For peer review only

Abstract

Survey research is sometimes regarded as an easy research approach. However, as with any other research approach and method, it is easy to conduct a survey of poor quality rather than one of high quality and real value. This paper provides a checklist of good practice in the conduct and reporting of survey research. Its purpose is to assist the novice researcher to produce survey work to a high standard, meaning a standard at which the results will be regarded as credible. The paper first provides an overview of the approach and then guides the reader step-by-step through the processes of data collection, data analysis, and reporting. It is not intended to provide a manual of how to conduct a survey, but rather to identify common pitfalls and oversights to be avoided by researchers if their work is to be valid and credible.

Keywords: data reporting, health care surveys, methodology, questionnaires, research design, survey methods, surveys

What is survey research?

Survey research is common in studies of health and health services, although its roots lie in the social surveys conducted in Victorian Britain by social reformers to collect information on poverty and working class life (e.g. Charles Booth [1] and Joseph Rowntree [2]), and indeed survey research remains most used in applied social research. The term 'survey' is used in a variety of ways, but generally refers to the selection of a relatively large sample of people from a pre-determined population (the 'population of interest'; this is the wider group of people in whom the researcher is interested in a particular study), followed by the collection of a relatively small amount of data from those individuals. The researcher therefore uses information from a sample of individuals to make some inference about the wider population.

Data are collected in a standardized form. This is usually, but not necessarily, done by means of a questionnaire or interview. Surveys are designed to provide a 'snapshot of how things are at a specific time' [3]. There is no attempt to control conditions or manipulate variables; surveys do not allocate participants into groups or vary the treatment they receive. Surveys are well suited to descriptive studies, but can also be used to explore aspects of a situation, or to seek explanation and provide data for testing hypotheses. It is important to recognize that 'the survey approach is a research strategy, not a research method' [3]. As with any research approach, a choice of methods is available and the one most appropriate to the individual project should be used. This paper will discuss the most popular methods employed in survey research, with an emphasis upon difficulties commonly encountered when using these methods.

Descriptive research

Descriptive research is a most basic type of enquiry that aims to observe (gather information on) certain phenomena, typically at a single point in time: the 'cross-sectional' survey. The aim is to examine a situation by describing important factors associated with that situation, such as demographic, socio-economic, and health characteristics, events, behaviours, attitudes, experiences, and knowledge. Descriptive studies are used to estimate specific parameters in a population (e.g. the prevalence of infant breast feeding) and to describe associations (e.g. the association between infant breast feeding and maternal age).

Analytical studies

Analytical studies go beyond simple description; their intention is to illuminate a specific problem through focused

Address reprint requests to John Sitzia, Research Department, Worthing Hospital, Lyndhurst Road, Worthing BN11 2DH, West Sussex, UK. E-mail: john.sitzia@wash.nhs.uk

© International Society for Quality in Health Care and Oxford University Press 2003; all rights reserved

For peer review only - http://bmjopen.bmj.com/site/about/guidelines.xhtml

data analysis, typically by looking at the effect of one set of variables upon another set. These are longitudinal studies, in which data are collected at more than one point in time with the aim of illuminating the direction of observed associations. Data may be collected from the same sample on each occasion (cohort or panel studies) or from a different sample at each point in time (trend studies).

Evaluation research

This form of research collects data to ascertain the effects of a planned change.

Advantages and disadvantages of survey research

Advantages:

- The research produces data based on real-world observations (empirical data).
- The breadth of coverage of many people or events means that it is more likely than some other approaches to obtain data based on a representative sample, and can therefore be generalizable to a population.
- Surveys can produce a large amount of data in a short time for a fairly low cost. Researchers can therefore set a finite time-span for a project, which can assist in planning and delivering end results.

Disadvantages:

- The significance of the data can become neglected if the researcher focuses too much on the range of coverage to the exclusion of an adequate account of the implications of those data for relevant issues, problems, or theories.
- The data that are produced are likely to lack details or depth on the topic being investigated.
- Securing a high response rate to a survey can be hard to control, particularly when it is carried out by post, but is also difficult when the survey is carried out face-to-face or over the telephone.

Essential steps in survey research

Research question

Good research has the characteristic that its purpose is to address a single clear and explicit research question; conversely, the end product of a study that aims to answer a number of diverse questions is often weak. Weakest of all, however, are those studies that have no research question at all and whose design simply is to collect a wide range of data and then to 'trawl' the data looking for 'interesting' or 'significant' associations. This is a trap novice researchers in particular fall into. Therefore, in developing a research question, the following aspects should be considered [4]:

- Be knowledgeable about the area you wish to research.
- Widen the base of your experience, explore related areas, and talk to other researchers and practitioners in the field you are surveying.
- Consider using techniques for enhancing creativity, for example brainstorming ideas.
- Avoid the pitfalls of: allowing a decision regarding methods to decide the questions to be asked; posing research questions that cannot be answered; asking questions that have already been answered satisfactorily.

Research methods

The survey approach can employ a range of methods to answer the research question. Common survey methods include postal questionnaires, face-to-face interviews, and telephone interviews.

Postal questionnaires

This method involves sending questionnaires to a large sample of people covering a wide geographical area. Postal questionnaires are usually received 'cold', without any previous contact between researcher and respondent. The response rate for this type of method is usually low, ~20%, depending on the content and length of the questionnaire. As response rates are low, a large sample is required when using postal questionnaires, for two main reasons: first, to ensure that the demographic profile of survey respondents reflects that of the survey population; and secondly, to provide a sufficiently large data set for analysis.

Face-to-face interviews

Face-to-face interviews involve the researcher approaching respondents personally, either in the street or by calling at people's homes. The researcher then asks the respondent a series of questions and notes their responses. The response rate is often higher than that of postal questionnaires as the researcher has the opportunity to sell the research to a potential respondent. Face-to-face interviewing is a more costly and time-consuming method than the postal survey, however the researcher can select the sample of respondents in order to balance the demographic profile of the sample.

Telephone interviews

Telephone surveys, like face-to-face interviews, allow a two-way interaction between researcher and respondent. Telephone surveys are quicker and cheaper than face-to-face interviewing. Whilst resulting in a higher response rate than postal surveys, telephone surveys often attract a higher level of refusals than face-to-face interviews as people feel less inhibited about refusing to take part when approached over the telephone.

Designing the research tool

Whether using a postal questionnaire or interview method, the questions asked have to be carefully planned and piloted.

For peer review only - http://bmjopen.bmj.com/site/about/guidelines.xhtml

The design, wording, form, and order of questions can affect the type of responses obtained, and careful design is needed to minimize bias in results. When designing a questionnaire or question route for interviewing, the following issues should be considered: (1) planning the content of a research tool; (2) questionnaire layout; (3) interview questions; (4) piloting; and (5) covering letter.

Planning the content of a research tool

The topics of interest should be carefully planned and relate clearly to the research question. It is often useful to involve experts in the field, colleagues, and members of the target population in question design in order to ensure the validity of the coverage of questions included in the tool (content validity).

Researchers should conduct a literature search to identify existing, psychometrically tested questionnaires. A well designed research tool is simple, appropriate for the intended use, acceptable to respondents, and should include a clear and interpretable scoring system. A research tool must also demonstrate the psychometric properties of reliability (consistency from one measurement to the next), validity (accurate measurement of the concept), and, if a longitudinal study, responsiveness to change [5]. The development of research tools, such as attitude scales, is a lengthy and costly process. It is important that researchers recognize that the development of the research tool is equal in importance—and deserves equal attention—to data collection. If a research instrument has not undergone a robust process of development and testing, the credibility of the research findings themselves may legitimately be called into question and may even be completely disregarded. Surveys of patient satisfaction and similar are commonly weak in this respect; one review found that only 6% of patient satisfaction studies used an instrument that had undergone even rudimentary testing [6]. Researchers who are unable or unwilling to undertake this process are strongly advised to consider adopting an existing, robust research tool.

Questionnaire layout

Questionnaires used in survey research should be clear and well presented. The use of capital (upper case) letters only should be avoided, as this format is hard to read. Questions should be numbered and clearly grouped by subject. Clear instructions should be given and headings included to make the questionnaire easier to follow.

The researcher must think about the form of the questions, avoiding 'double-barrelled' questions (two or more questions in one, e.g. 'How satisfied were you with your personal nurse and the nurses in general?'), questions containing double negatives, and leading or ambiguous questions. Questions may be open (where the respondent composes the reply) or closed (where pre-coded response options are available, e.g. multiple-choice questions). Closed questions with pre-coded response options are most suitable for topics where the possible responses are known. Closed questions are quick to administer and can be easily coded and analysed. Open questions should be used where possible replies are unknown or too numerous to pre-code. Open questions are more demanding for respondents but if well answered can provide useful insight into a topic. Open questions, however, can be time consuming to administer and difficult to analyse. Whether using open or closed questions, researchers should plan clearly how answers will be analysed.

Interview questions

Open questions are used more frequently in unstructured interviews, whereas closed questions typically appear in structured interview schedules. A structured interview is like a questionnaire that is administered face to face with the respondent. When designing the questions for a structured interview, the researcher should consider the points highlighted above regarding questionnaires. The interviewer should have a standardized list of questions, each respondent being asked the same questions in the same order. If closed questions are used the interviewer should also have a range of pre-coded responses available.

If carrying out a semi-structured interview, the researcher should have a clear, well thought out set of questions; however, the questions may take an open form and the researcher may vary the order in which topics are considered.

Piloting

A research tool should be tested on a pilot sample of members of the target population. This process will allow the researcher to identify whether respondents understand the questions and instructions, and whether the meaning of questions is the same for all respondents. Where closed questions are used, piloting will highlight whether sufficient response categories are available, and whether any questions are systematically missed by respondents.

When conducting a pilot, the same procedure as as that to be used in the main survey should be followed; this will highlight potential problems such as poor response.

Covering letter

All participants should be given a covering letter including information such as the organization behind the study, including the contact name and address of the researcher, details of how and why the respondent was selected, the aims of the study, any potential benefits or harm resulting from the study, and what will happen to the information provided. The covering letter should both encourage the respondent to participate in the study and also meet the requirements of informed consent (see below).

Sample and sampling

The concept of sample is intrinsic to survey research. Usually, it is impractical and uneconomical to collect data from every single person in a given population; a sample of the population has to be selected [7]. This is illustrated in the following

For peer review only - http://bmjopen.bmj.com/site/about/guidelines.xhtml

hypothetical example. A hospital wants to conduct a satisfaction survey of the 1000 patients discharged in the previous month; however, as it is too costly to survey each patient, a sample has to be selected. In this example, the researcher will have a list of the population members to be surveyed (sampling frame). It is important to ensure that this list is both up-to date and has been obtained from a reliable source.

The method by which the sample is selected from a sampling frame is integral to the external validity of a survey: the sample has to be representative of the larger population to obtain a composite profile of that population [8].

There are methodological factors to consider when deciding who will be in a sample: How will the sample be selected? What is the optimal sample size to minimize sampling error? How can response rates be maximized?

The survey methods discussed below influence how a sample is selected and the size of the sample. There are two categories of sampling: random and non-random sampling, with a number of sampling selection techniques contained within the two categories. The principal techniques are described here [9].

Random sampling

Generally, random sampling is employed when quantitative methods are used to collect data (e.g. questionnaires). Random sampling allows the results to be generalized to the larger population and statistical analysis performed if appropriate. The most stringent technique is simple random sampling. Using this technique, each individual within the chosen population is selected by chance and is equally as likely to be picked as anyone else. Referring back to the hypothetical example, each patient is given a serial identifier and then an appropriate number of the 1000 population members are randomly selected. This is best done using a random number table, which can be generated using computer software (a free on-line randomizer can be found at http://www.randomizer.org/index.htm).

Alternative random sampling techniques are briefly described. In systematic sampling, individuals to be included in the sample are chosen at equal intervals from the population; using the earlier example, every fifth patient discharged from hospital would be included in the survey. Stratified sampling selects a specific group and then a random sample is selected. Using our example, the hospital may decide only to survey older surgical patients. Bigger surveys may employ cluster sampling, which randomly assigns groups from a large population and then surveys everyone within the groups, a technique often used in national-scale studies.

Non-random sampling

Non-random sampling is commonly applied when qualitative methods (e.g. focus groups and interviews) are used to collect data, and is typically used for exploratory work. Non-random sampling deliberately targets individuals within a population. There are three main techniques. (1) purposive sampling: a specific population is identified and only its members are included in the survey; using our example above, the hospital may decide to survey only patients who had an appendectomy. (2) Convenience sampling: the sample is made up of the individuals who are the easiest to recruit. Finally, (3) snowballing: the sample is identified as the survey progresses; as one individual is surveyed he or she is invited to recommend others to be surveyed.

It is important to use the right method of sampling and to be aware of the limitations and statistical implications of each. The need to ensure that the sample is representative of the larger population was highlighted earlier and, alongside the sampling method, the degree of sampling error should be considered. Sampling error is the probability that any one sample is not completely representative of the population from which it has been drawn [9]. Although sampling error cannot be eliminated entirely, the sampling technique chosen will influence the extent of the error. Simple random sampling will give a closer estimate of the population than a convenience sample of individuals who just happened to be in the right place at the right time.

Sample size

What sample size is required for a survey? There is no definitive answer to this question: large samples with rigorous selection are more powerful as they will yield more accurate results, but data collection and analysis will be proportionately more time consuming and expensive. Essentially, the target sample size for a survey depends on three main factors: the resources available, the aim of the study, and the statistical quality needed for the survey. For 'qualitative' surveys using focus groups or interviews, the sample size needed will be smaller than if quantitative data is collected by questionnaire. If statistical analysis is to be performed on the data then sample size calculations should be conducted. This can be done using computer packages such as G^{*}Power [10]; however, those with little statistical knowledge should consult a statistician. For practical recommendations on sample size, the set of survey guidelines developed by the UK Department of Health [11] should be consulted.

Larger samples give a better estimate of the population but it can be difficult to obtain an adequate number of responses. It is rare that everyone asked to participate in the survey will reply. To ensure a sufficient number of responses, include an estimated non-response rate in the sample size calculations.

Response rates are a potential source of bias. The results from a survey with a large non-response rate could be misleading and only representative of those who replied. French [12] reported that non-responders to patient satisfaction surveys are less likely to be satisfied than people who reply. It is unwise to define a level above which a response rate is acceptable, as this depends on many local factors; however, an achievable and acceptable rate is ~75% for interviews and 65% for self-completion postal questionnaires [9,13]. In any study, the final response rate should be reported with the results; potential differences between the respondents and non-respondents should be explicitly explored and their implications discussed.

For peer review only - http://bmjopen.bmj.com/site/about/guidelines.xhtml

There are techniques to increase response rates. A questionnaire must be concise and easy to understand, reminders should be sent out, and method of recruitment should be carefully considered. Sitzia and Wood [13] found that participants recruited by mail or who had to respond by mail had a lower mean response rate (67%) than participants who were recruited personally (mean response 76.7%). A most useful review of methods to maximize response rates in postal surveys has recently been published [14].

Data collection

Researchers should approach data collection in a rigorous and ethical manner. The following information must be clearly recorded:

- How, where, how many times, and by whom potential respondents were contacted.
- How many people were approached and how many of those agreed to participate.
- How did those who agreed to participate differ from those who refused with regard to characteristics of interest in the study, for example how were they identified, where were they approached, and what was their gender, age, and features of their illness or health care.
- How was the survey administered (e.g. telephone interview).
- What was the response rate (i.e. the number of usable data sets as a proportion of the number of people approached).

Data analysis

The purpose of all analyses is to summarize data so that it is easily understood and provides the answers to our original questions: 'In order to do this researchers must carefully examine their data; they should become friends with their data' [15]. Researchers must prepare to spend substantial time on the data analysis phase of a survey (and this should be built into the project plan). When analysis is rushed, often important aspects of the data are missed and sometimes the wrong analyses are conducted, leading to both inaccurate results and misleading conclusions [16]. However, and this point cannot be stressed strongly enough, researchers must not engage in data dredging, a practice that can arise especially in studies in which large numbers of dependent variables can be related to large numbers of independent variables (outcomes). When large numbers of possible associations in a dataset are reviewed at $P < 0.05$, one in 20 of the associations by chance will appear 'statistically significant'; in datasets where only a few real associations exist, testing at this significance level will result in the large majority of findings still being false positives [17].

The method of data analysis will depend on the design of the survey and should have been carefully considered in the planning stages of the survey. Data collected by qualitative methods should be analysed using established methods such as content analysis [18], and where quantitative methods have been used appropriate statistical tests can be applied. Describing methods of analysis here would be unproductive as a multitude of introductory textbooks and on-line resources are available to help with simple analyses of data (e.g. [19, 20]). For advanced analysis a statistician should be consulted.

Reporting

When reporting survey research, it is essential that a number of key points are covered (though the length and depth of reporting will be dependent upon journal style). These key points are presented as a 'checklist' below:

(1) Explain the purpose or aim of the research, with the explicit identification of the research question.
(2) Explain why the research was necessary and place the study in context, drawing upon previous work in relevant fields (the literature review).
(3) Describe in (proportionate) detail how the research was done.
  (a) State the chosen research method or methods, and justify why this method was chosen.
  (b) Describe the research tool. If an existing tool is used, briefly state its psychometric properties and provide references to the original development work. If a new tool is used, you should include an entire section describing the steps undertaken to develop and test the tool, including results of psychometric testing.
  (c) Describe how the sample was selected and how data were collected, including:
    (i) How were potential subjects identified?
    (ii) How many and what type of attempts were made to contact subjects?
    (iii) Who approached potential subjects?
    (iv) Where were potential subjects approached?
    (v) How was informed consent obtained?
    (vi) How many agreed to participate?
    (vii) How did those who agreed differ from those who did not agree?
    (viii) What was the response rate?
(4) Describe and justify the methods and tests used for data analysis.
(5) Present the results of the research. The results section should be clear, factual, and concise.
(6) Interpret and discuss the findings. This 'discussion' section should not simply reiterate results; it should provide the author's critical reflection upon both the results and the processes of data collection. The discussion should assess how well the study met the research question, should describe the problems encountered in the research, and should honestly judge the limitations of the work.
(7) Present conclusions and recommendations.

The researcher needs to tailor the research report to meet:

For peer review only - http://bmjopen.bmj.com/site/about/guidelines.xhtml

- The expectations of the specific audience for whom the work is being written.
- The conventions that operate at a general level with respect to the production of reports on research in the social sciences.

Ethics

Anyone involved in collecting data from patients has an ethical duty to respect each individual participant's autonomy. Any survey should be conducted in an ethical manner and one that accords with best research practice. Two important ethical issues to adhere to when conducting a survey are confidentiality and informed consent.

The respondent's right to confidentiality should always be respected and any legal requirements on data protection adhered to. In the majority of surveys, the patient should be fully informed about the aims of the survey, and the patient's consent to participate in the survey must be obtained and recorded.

The professional bodies listed below, among many others, provide guidance on the ethical conduct of research and surveys.

- American Psychological Association: http://www.apa.org
- British Psychological Society: http://www.bps.org.uk
- British Medical Association: http://www.bma.org.uk.
- UK General Medical Council: http://www.gmc-uk.org
- American Medical Association: http://www.ama-assn.org
- UK Royal College of Nursing: http://www.rcn.org.uk
- UK Department of Health: http://www.doh.gov

Conclusion

Survey research demands the same standards in research practice as any other research approach, and journal editors and the broader research community will judge a report of survey research with the same level of rigour as any other research report. This is not to say that survey research need be particularly difficult or complex; the point to emphasize is that researchers should be aware of the steps required in survey research, and should be systematic and thoughtful in the planning, execution, and reporting of the project. Above all, survey research should not be seen as an easy, 'quick and dirty' option; such work may adequately fulfil local needs (e.g. a quick survey of hospital staff satisfaction), but will not stand up to academic scrutiny and will not be regarded as having much value as a contribution to knowledge.

References

1. London School of Economics, UK. Http://booth.lse.ac.uk/ (accessed 15 January 2003).
2. Vernon A. *A Quaker Businessman: Biography of Joseph Rowntree (1836–1925)*. London: Allen & Unwin, 1958.
3. Denscombe M. *The Good Research Guide: For Small-scale Social Research Projects*. Buckingham: Open University Press, 1998.
4. Robson C. *Real World Research: A Resource for Social Scientists and Practitioner-researchers*. Oxford: Blackwell Publishers, 1993.
5. Streiner DL, Norman GR. *Health Measurement Scales: A Practical Guide to their Development and Use*. Oxford: Oxford University Press, 1995.
6. Sitzia J. How valid and reliable are patient satisfaction data? An analysis of 195 studies. *Int J Qual Health Care* 1999; **11:** 319–328.
7. Bowling A. *Research Methods in Health. Investigating Health and Health Services*. Buckingham: Open University Press, 2002.
8. American Statistical Association, USA. Http://www.amstat.org (accessed 9 December 2002).
9. Arber S. Designing samples. In: Gilbert N, ed. *Researching Social Life*. London: SAGE Publications, 2001.
10. Heinrich Heine University, Dusseldorf, Germany. Http://www.psycho.uni-duesseldorf.de/aap/projects/gpower/index.html (accessed 12 December 2002).
11. Department of Health, England. Http://www.doh.gov.uk/acutesurvey/index.htm (accessed 12 December 2002).
12. French K. Methodological considerations in hospital patient opinion surveys. *Int J Nurs Stud* 1981; **18:** 7–32.
13. Sitzia J, Wood N. Response rate in patient satisfaction research: an analysis of 210 published studies. *Int J Qual Health Care* 1998; **10:** 311–317.
14. Edwards P, Roberts I, Clarke M *et al.* Increasing response rates to postal questionnaires: systematic review. *Br Med J* 2002; **324:** 1183.
15. Wright DB. Making friends with our data: improving how statistical results are reported. *Br J Educ Psychol* 2003; in press.
16. Wright DB, Kelley K. Analysing and reporting data. In: Michie S, Abraham C, eds. *Health Psychology in Practice*. London: SAGE Publications, 2003; in press.
17. Davey Smith G, Ebrahim S. Data dredging, bias, or confounding. *Br Med J* 2002; **325:** 1437–1438.
18. Morse JM, Field PA. *Nursing Research: The Application of Qualitative Approaches*. London: Chapman and Hall, 1996.
19. Wright DB. *Understanding Statistics: An Introduction for the Social Sciences*. London: SAGE Publications, 1997.
20. Sportscience, New Zealand. Http://www.sportsci.org/resource/stats/index.html (accessed 12 December 2002).

Accepted for publication 16 January 2003

For peer review only - http://bmjopen.bmj.com/site/about/guidelines.xhtml

BMJ Open

Attitudes of physicians towards target groups and content of the discharge summary– a cross-sectional analysis in Styria, Austria

Journal:	*BMJ Open*
Manuscript ID	bmjopen-2019-034857.R1
Article Type:	Original research
Date Submitted by the Author:	18-Nov-2019
Complete List of Authors:	Hoffmann, Magdalena; Hospital of the Federal State of Styria and University Hospital Graz, Executive Department for Quality and Risk Management ; Medical University of Graz, Research Unit for Safety in Health, Division of Plastic, Aesthetic and Reconstructive Surgery, Department of Surgery<br>Schwarz, Christine; Hospital of the Federal State of Styria and University Hospital Graz, Executive Department for Quality and Risk Management ; Medical University of Graz, Research Unit for Safety in Health, Division of Plastic, Aesthetic and Reconstructive Surgery, Department of Surgery<br>Pregartner, Gudrun; Medical University of Graz, Institute for Medical Informatics, Statistics und Documentation<br>Weinrauch, Maximilian; Medical University of Graz<br>Jantscher, Lydia ; Hospital of the Federal State of Styria and University Hospital Graz, Executive Department for Quality and Risk Management<br>Kamolz, Lars; Medical University of Graz, Research Unit for Safety in Health, Division of Plastic, Aesthetic and Reconstructive Surgery, Department of Surgery<br>Brunner, Gernot; Medical University of Graz, Research Unit for Safety in Health, Division of Plastic, Aesthetic and Reconstructive Surgery, Department of Surgery<br>Sendlhofer, Gerald; Hospital of the Federal State of Styria and University Hospital Graz, Executive Department for Quality and Risk Management; Medical University of Graz, Research Unit for Safety in Health, Division of Plastic, Aesthetic and Reconstructive Surgery, Department of Surgery
**Primary Subject Heading**:	Health services research
Secondary Subject Heading:	General practice / Family practice
Keywords:	GENERAL MEDICINE (see Internal Medicine), HEALTH SERVICES ADMINISTRATION & MANAGEMENT, Health & safety < HEALTH SERVICES ADMINISTRATION & MANAGEMENT, Quality in health care < HEALTH SERVICES ADMINISTRATION & MANAGEMENT

For peer review only - http://bmjopen.bmj.com/site/about/guidelines.xhtml

SCHOLARONE™
Manuscripts

Attitudes of physicians towards target groups and content of the discharge summary– a cross-sectional analysis in Styria, Austria

Magdalena Hoffmann[1,2,3], Christine Maria Schwarz[1,2*], Gudrun Pregartner[4], Maximilian Weinrauch[5], Lydia Jantscher[1], Lars-Peter Kamolz[2], Gernot Brunner[2], Gerald Sendlhofer[1,2]

Author Affiliations:

[1]Executive Department for Quality and Risk Management, University Hospital Graz, Austria

[2]Research Unit for Safety in Health, Division of Plastic, Aesthetic and Reconstructive Surgery, Department of Surgery, Medical University of Graz, Austria

[3]Division of Endocrinology and Diabetology, Department of Internal Medicine, Medical University of Graz, Austria

[4]Institute for Medical Informatics, Statistics und Documentation, Medical University of Graz, Austria

[5]Medical University of Graz, Austria

<u>* Corresponding author:</u>

Christine Schwarz, MSc, BSc, Research Unit for Safety in Health, c/o Division of Plastic, Aesthetic and Reconstructive Surgery, Department of Surgery, Medical University of Graz, Auenbruggerplatz 1/3, 8036 Graz, Austria, Tel +43664 1188128, Email: Christine.schwarz@medunigraz.at

For peer review only - http://bmjopen.bmj.com/site/about/guidelines.xhtml

First author

Magdalena Hoffmann, MBA, MSc, MSc, PhD magdalena.hoffmann@medunigraz.at

Co-autors

Christine Maria Schwarz, MSc, BSc: Christine.schwarz@medunigraz.at

Gudrun Pregartner, MSc, BSc: Gudrun.pregartner@medunigraz.at

Maximilian Weinrauch, MD: Maximilian.weinrauch@stud.medunigraz.at

Lydia Jantscher, B.A: Lydia.jantscher@klinikum-graz.at

Lars-Peter Kamolz, MD, MSc, PhD: Lars.kamolz@medunigraz.at

Gernot Brunner, MD, MSc: Gernot.brunner@medunigraz.at

Gerald Sendlhofer PhD, MSc: Gerald.Sendlhofer@klinikum-graz.at

Short title: Attitudes of physicians concerning the discharge summary

Word count: Abstract (234); Text (2676)

Number of tables: 5

Number of figures: 0

Keywords: Patients, discharge summary, risk, physicians, health literacy

For peer review only

Strengths and limitations of this study:

- This work investigated the question for whom the discharge summary is to rethink a historical grown attitude.
- A strengths of this study is the total amount of participating physicians (n=1060).
- The discharge summary is primarily considered as a communication tool among physicians; however, patients are also indicated as addressees.
- A limitations of the study is the moderate response rate of external physicians as well as the sample selection, which does not include private/spiritual hospitals or other regions in Austria.

For peer review only

Abstract

Objectives: The discharge summary represents one of the most important instruments to ensure a safe patient discharge from the hospital. They sometimes have poor quality in content and often include medical jargon, which the patient and their relatives cannot easily understand. Therefore, many risks for patient safety exist. This study investigated the questions for whom the discharge summary is and which contents are necessary to ensure a safe treatment.

Design: cross-sectional analysis

Setting: Styria, Austria

Participants: 3948 internal and external physicians were consulted

Interventions: An online survey consisting of 24 questions was conducted. The survey was distributed to physicians working in the province of Styria, Austria, in 2018 over a period of 6 months.

Main Outcomes and Measures: Attitudes of internal and external physicians in terms of target group, content and health literacy

Results: In total, 1060 physicians participated in the survey. The discharge summary is considered as a communication tool among physicians (97.9%) and the patients are also indicated as addressees (73.5%). Furthermore, there is a high level of agreement that understandable information in the discharge summary leads to fewer questions of the patients (67.9%).

Conclusion: In conclusion, the discharge summary is not only seen as a document for the further treating physician but is also relevant for the patient. Incorporating the patient into their treatment at all levels may possibly strengthen the individual health literacy of the patient and their caring relatives.

For peer review only - http://bmjopen.bmj.com/site/about/guidelines.xhtml

For peer review only

Trial registration: The study was approved by the Medical University Graz Ethics Committee (EK 30-220 ex 17/18).

For peer review only

For peer review only - http://bmjopen.bmj.com/site/about/guidelines.xhtml

Introduction

In order to ensure a safe patient discharge from the hospital in terms of offering all relevant information, the discharge summary (DS) represents one of the most important instruments for this purpose. It usually contains diagnosis, recommendations for further therapy and treatment, monitoring appointments as well as other important information for the therapy of patients. A written DS is legally required and necessary because patients often struggle to accurately recall medical information given by the physician, especially when they are old or anxious. Patients tend to focus on diagnosis-related information and fail to register treatment instructions (1). Therefore, the DS should be delivered to the patient and general practitioner (GP) to ensure a continuous treatment of the patient after discharge from hospital (2). In practice, DS are often of poor quality in terms of information content and/or utilise medical jargon such as unexplained abbreviations of medical terms, which the patient and their relatives and even GP struggle to comprehend (3,4). Choudhry *et al.* (2016) reported that for example in Rochester, United States of America, trauma patient DS are written at a too advanced educational level (5). Problematic consequences for the treatment continuity can be manifold if the comprehensive medical language isn‘t adequately understood.

The European Health Literacy Survey report 2012 indicates that in the Austrian population the health literacy is below European Union average (6). In addition, DS in Austria are not governed by detailed national standards for content or structure so far. Therefore, hospitals generated their own format and selected specific contents. The legal structure of the electronic health record (ELGA), which also requires a standardisation of DS by law since 2015, is not yet widespread in Austria. The structure specifications of ELGA are only a rough guide for headings (e.g. diagnosis, outcome measurement, medication, summary) in a given order; however, there are hardly any specific contents offered (7).

For peer review only - http://bmjopen.bmj.com/site/about/guidelines.xhtml

Furthermore, the lack of a uniform structure and of important contents in the DS is an international issue as two studies of patients with kidney disease have shown (8,9).

Recent studies reported that patients and their relatives actively want to participate in their care and most patients want to understand their DS (10). It is crucial that patients receive easily comprehensible written medical information as well as therapeutically relevant care information in a patient-directed DS. Lin *et al*. (2014) showed in a prospective randomised controlled trial that a simple patient-directed DS delivered during a brief discussion at discharge significantly improved the patient's understanding of their illness and post-discharge recommendations, which were vice versa limited (11). Patient-directed DS are intended to increase patient understanding as well as to improve the doctor- patient relationship (12,13). A multitude of positive effects, such as increased patient satisfaction (14), increased understanding, as well as a greater patient involvement in their care, were shown (15).

The aim of this study was to investigate attitudes and perceptions among physicians in Styria regarding the current DS in terms of who the addressee of the DS is and what the necessary content is to improve the communication from hospital to the further treating GP or other health care workers in the future. The study was carried out by the University Hospital Graz, Austria, and involved physicians in and outside of the University hospital.

Methods

Ethics statement

The Ethics Committee of the Medical University of Graz approved the study (vote#: 30-220 ex17/18).

Reporting

For peer review only - http://bmjopen.bmj.com/site/about/guidelines.xhtml

The research and reporting methodology followed by “Recruitment process and description of the sample having access to the questionnaire” (16) recommended by equator network.

The survey

This cross-sectional survey was based on a literature review about challenges of the medical DS (3) and contained 24 questions on specific topics (e.g. target group, necessary content, contribution to health literacy, need of change) related to the DS. Each item was scored on a four-point Likert-type scale from 1 – totally agree – to 4 – totally disagree – and provided the additional option of ‘not relevant’. After a review process of medical professionals, the survey was pre-tested in 10 individuals (nurses, physicians, and staff from the quality and risk management department) several times. A detailed description of the survey is presented in **TABLE 1.** The online survey was distributed via EvaSys (Electric Paper Evaluationssysteme GmbH, Germany, version 7.1) in 2018 over a period of 6 months.

Study Population

Two groups of physicians, those working in public Styrian hospitals (Styrian Hospitals Limited Liability Company (KAGes)) as well as physicians working outside as a consultant in the province of Styria, were asked to complete the survey. The groups are referred to as “internal” and “external”, respectively. The internal group consisted of 2387 physicians and the external group of 1561 physicians. Both groups were invited via Email to the survey. Three reminders within four weeks were sent to non-responders.

Statistical analysis

The survey data were pooled into the categories “agree” (scores 1/2) and “disagree” (scores 3/4) and were descriptively analysed using absolute and relative frequencies. Differences between the two

For peer review only - http://bmjopen.bmj.com/site/about/guidelines.xhtml

physician groups as well as for gender and years of work experience were determined by Fisher's exact test in an exploratory fashion and a p-value of <0.05 was considered significant. When comparing more than two groups, post-hoc tests were performed and a Bonferroni correction to the significance level was applied. For three pairwise comparisons a p value <0.017 was thus considered significant. All analyses were performed using R version 3.5.3.

Patient and public involvement

This questionnaire was developed from results of preliminary work with focus group discussions with patients, experts and stakeholders however; this present research was done without patient involvement. Our preliminary results were presented on a science to public congress for further discussion.

Results

In total, 1060 physicians participated in the survey; 747 internal physicians (response rate 31%) and 313 external physicians (response rate 20%) provided answers. For each of the 24 questions of the survey, a minimum participation rate of 88% was observed, i.e. the respondent answered with one of the four intended answer categories. A detailed description of the participating physicians is presented in **TABLE 2**.

Overall survey results

Although the DS is primarily considered as a communication tool among physicians (97.9%), patients were also indicated as addressees (73.5%). Furthermore, two thirds of the respondents indicated that easily comprehensible information leads to fewer questions from the patients (67.9%). Regarding the

For peer review only - http://bmjopen.bmj.com/site/about/guidelines.xhtml

necessary content of the DS, diagnosis (100%), therapy (99.7%), recommendations on further treatment (99.6%), control visits and follow-up appointments (98.7%), prescription of medication (98.5%), and behavioural recommendations for the patients (94.4%) receive most agreement from the respondents. Only less than a quarter of participants (22.5%) wish to have specific abbreviations in their DS.

The DS in its current form is felt to contribute only mediocrely to health literacy (49.4%), and most respondents are of the opinion that changes should be made (46.2%). A detailed description of the physicians' ratings is presented in **TABLE 3**.

Differences between internal and external physicians

There were significant differences given by internal and external physicians for seven of the 24 items. Patients were more commonly considered as addressees of the DS by the internal group (75.8% versus 67.8%, p=0.009). The internal group was less frequently of the opinion that a DS that is comprehensible for patients leads to less time-consuming questions (65.8% versus 72.7%, p=0.034) and that patients need not understand the DS (23.9% versus 32.3%, p=0.006). Additionally, less internal physicians received a training in how a DS should be structured (46.8% versus 55.5%, p=0.012). Internal physicians were more often of the opinion that the content of the DS in its current form is sufficient for further treatment (81.0% versus 71.5%, p=0.001) and that the DS contributes to increase the individual health literacy of patients (52.8% versus 41.2%, p=0.001). Whereas less than half of the internal group feels the need to make changes to the current DS, more than half of the external group would like to see changes made (41.4% versus 57.9%, p<0.001). A detailed description of the physicians' ratings is presented in **TABLE 3.**

For peer review only - http://bmjopen.bmj.com/site/about/guidelines.xhtml

Differences according to gender

Four-hundred sixty (43.4%) of the respondents were female, 565 (53.3%) were male, and 35 (3.3%) did not disclose their gender. Female physicians more often see patients (82.2% versus 67.0%, $p<0.001$) and all persons authorized by the patient (70.4% versus 60.9%, $p=0.002$) as addressees of the DS. More of them than their male colleagues also believe that a DS that is understandable for patients leads to less time-consuming questions (71.7% versus 65.0%, $p=0.029$). Conversely, less female physicians agree with that the patient need not understand the DS (18.9% versus 31.8%, $p<0.001$) or that abbreviations are a necessary part of the DS (17.2% versus 26.0%, $p=0.001$). Interestingly, more female physicians want therapy recommendations or reports from other health care professionals, such as clinical social workers (59.5% versus 51.1%, $p=0.010$) or medical-technical services (72.0% versus 65.0%, $p=0.022$), to be included in the DS. A detailed description of the responses by gender is presented in **TABLE 4.**

Differences according to years of work experience

Three-hundred nine (29.2%) of the respondents had a work experience of up to ten years, 274 (25.8%) had been working for 11 to 20 years, and 461 (43.5%) for more than 20 years; only 16 (1.5%) did not disclose this information. Physicians with up to 20 years of work experience see the patient more often as addressee of the DS than those who have been working in the field longer (80.5% of physicians with a work experience of 0-10 years versus 79.3% of those working 11-20 years versus 64% of those working >20 years, $p<0.001$). Physicians with less professional experience also tend to agree less with the statement that the DS does not need to be understood by patients (16.7% versus

For peer review only - http://bmjopen.bmj.com/site/about/guidelines.xhtml

23.5% versus 34.6%, p<0.001). The groups also showed different rates of agreement to the statement that, in future, the DS should contain further procedures and treatment goals in an understandable way, so that patients themselves can contribute to the improvement of their health (81.1% versus 84.2% versus 75.2%, p=0.011). Physicians with more work experience were more open to changes of the DS than younger ones (39.3% versus 49.0% versus 49.6%, p=0.016). A detailed description of the responses by work experience is presented in **TABLE 5.**

Discussion

The main finding of this cross-sectional survey is that physicians consider themselves as the target group of the DS. The physicians have a clear view regarding the required content in the DS. However, gender and years of work experience also influence the results.

Implications of findings

This survey showed that most physicians consider themselves as the target group of the DS; however, patients, who according to the Austrian law are the owners of the written DS (17), are also often indicated as a target group. Patient-comprehensible DS support patients and relatives in understanding medical information as they include the most important messages when leaving the hospital. A very early study by Shankar in 2003 stated that simple and specific instructions for patients are better recalled than general statements. Patient comprehension can be aided with written or visual material (1). Health literacy means finding, understanding, and using health information (18). However, fewer than half of physicians said that the current DS supports health literacy of patients. A patient-directed DS could support finding the right information and understanding the offered health information (10,19,20). Positive effects of a patient-directed DS were reported by Lin

For peer review only - http://bmjopen.bmj.com/site/about/guidelines.xhtml

et al. in 2014. Patients who received a patient-directed DS had an immediate increase in the understanding of tests performed at the hospital as well as a higher compliance in post-discharge recommendations (11). Wernick *et al.* reported in 2016 that minimising the use of medical terminology in medical correspondence significantly improved patient understanding and perception of their ability to manage chronic health conditions (16). Our results also showed that the majority of physicians consider medical terminology a necessary part of DS but disapprove the use of abbreviations.

Our results show that physicians have a clear expectation regarding the required content in the DS. The need for specific content in Styria is similar to other studies (21). The use of ELGA criteria, providing a minimum number of (mandatory and optional) headings in a fixed structure, to guide physicians in writing a comprehensive DS holds promise in improving the existing deficits in communication at transitions of care, as another study using a standardized DS already showed (14).

For a majority of physicians, abbreviations are undesirable since they are not only problematic for patients but also for physicians. Chemali *et al.* reported in 2015 that abbreviations used in hospital DS are not well understood by the GPs who receive them (17). In another study of Shilo *et al.* abbreviations from 80 DS and admission notes from orthopaedic surgery and medical wards were extracted and graded by senior physicians as understandable, ambiguous, or unknown (22). To improve comprehension of the DS the use of (ambiguous and unknown) abbreviations should be decreased.

It is noteworthy that female physicians expressed that the DS should contain more reports from other health care workers. Furthermore, they see a greater need for patients to understand the DS than male physicians do. The differences in answers related to the years of work experience may indicate a paradigm shift. Physicians with fewer years of experience see the patient significantly more often as

For peer review only - http://bmjopen.bmj.com/site/about/guidelines.xhtml

addressee of the DS compared to physicians with more than 20 years of work experience. The authors are not aware of comparable findings from current literature.

Our results showed that less than half of the physicians had received training to write the DS. However, physicians with more years of work experience more frequently stated to have received specific training as part of their education. In a study by Yemm *et al*. in 2014, 36% of young physicians reported that they had inadequate training in composing a DS (15). Therefore, it may be an advantage for young medical professionals to have a fixed structure and guidelines for its content. However, a good structure and layout does not replace the training in writing medical DS. A study by Shivji *et al*. has demonstrated that educational sessions improved the quality of written DS (23). We suggest that a course in writing DS should be included as part of the curriculum in medical schools, as it is important for junior doctors to learn how to prepare a DS.

Furthermore, to improve the DS, supporting instruments and standards need to be implemented. In 2018 Savvopoulos *et al*. developed a scoring tool to assess the quality of the DS (16). Such tools can help to assess and improve the quality of current DS and derive any need for improvement if necessary.

Strengths and limitations of the study

A strength of this study is the high response rate of internal physicians and the involvement of occupational groups and patients in the project process before and after the survey.

This study has several limitations. This survey is a snapshot of current attitudes of doctors regarding discharge information. Another limitation are the dropped non-responders of the survey. A limitation of the present study is the sample selection, which does not include private/spiritual hospitals or other

For peer review only

For peer review only - http://bmjopen.bmj.com/site/about/guidelines.xhtml

regions in Austria. Another important limitation of our study is the moderate response rate of external physicians that constitutes a potential bias. However, this phenomenon was also observed in other surveys with externals. It might be that external physicians can hardly be attracted for participation.

Conclusions

Based on our results, it can be concluded that the DS is perceived not only as a document for the further treating physician but also for the patient and other caregivers. Now, the discharge summary primarily is written for the further treating doctor and has often not a uniform structure. The question arises how a discharge summary can be designed, that is partly understandable for the patient and relatives and which can be implemented in the clinic practice. Further research on this topic should address this question. An international comparison of physicians' perceptions, attitudes, and solutions regarding a patient-directed DS, important contents, and improvements in health literacy of patients would be beneficial.

For peer review only

References

1. Kessels RPC. Patients' memory for medical information. J R Soc Med [Internet]. 2003 May [cited 2019 Jul 26];96(5):219–22. Available from: http://www.ncbi.nlm.nih.gov/pubmed/12724430

2. BMASGK. Zielsteuerung-Gesundheit Qualitätsstandard Aufnahme-und Entlassungs-management [Internet]. 2018 [cited 2019 May 24]. Available from: https://www.sozialministerium.at/cms/site/attachments/1/3/0/CH3970/CMS1350910195632/qualitaetsstandard_aufem.pdf

3. Schwarz CM, Hoffmann M, Schwarz P, Kamolz L, Brunner G, Sendlhofer G. A systematic literature review and narrative synthesis on the risks of medical discharge letters for patients ' safety. 2019;1:1–10.

4. Sarzynski E, Hashmi H, Subramanian J, Fitzpatrick L, Polverento M, Simmons M, et al. Opportunities to improve clinical summaries for patients at hospital discharge. BMJ Qual Saf [Internet]. 2016 May 6 [cited 2019 Jul 26];26(5):bmjqs-2015-005201. Available from: http://www.ncbi.nlm.nih.gov/pubmed/27154878

5. Choudhry AJ, Baghdadi YMK, Wagie AE, Habermann EB, Heller SF, Jenkins DH, et al. Readability of discharge summaries: With what level of information are we dismissing our patients? In: American Journal of Surgery. 2016. p. 631–6.

6. Sorensen K, Pelikan JM, Rothlin F, Ganahl K, Slonska Z, Doyle G, et al. Health literacy in Europe: comparative results of the European health literacy survey (HLS-EU). Eur J Public Health. 2015;25(6):1–6.

For peer review only

For peer review only - http://bmjopen.bmj.com/site/about/guidelines.xhtml

7. Sabutsch S, Brandstätter J. ELGA CDA Implementierungsleitfäden. 2013;1–216. Available from: https://www.gesundheit.gv.at/Portal.Node/ghp/public/content/ELGA/elga-was-ist-elga.html

8. Harel Z, Wald R, Perl J, Schwartz D, Bell CM. Evaluation of deficiencies in current discharge summaries for dialysis patients in Canada. J Multidiscip Healthc. 2012;5:77–84.

9. Greer RC, Liu Y, Crews DC, Jaar BG, Rabb H, Boulware LE. Hospital discharge communications during care transitions for patients with acute kidney injury: a cross-sectional study. BMC Health Serv Res [Internet]. 2016 Dec 30 [cited 2019 Aug 1];16(1):449. Available from: http://www.ncbi.nlm.nih.gov/pubmed/27577888

10. Hammerer P. Patientenverständliche Arztbriefe und Befunde. Springer Medizin. 2018;

11. Lin R, Gallagher R, Spinaze M, Najoumian H, Dennis C, Clifton-Bligh R, et al. Effect of a patient-directed discharge letter on patient understanding of their hospitalisation. Intern Med J [Internet]. 2014 Sep 1 [cited 2018 Jan 29];44(9):851–7. Available from: http://doi.wiley.com/10.1111/imj.12482

12. White P, Singleton A, Jones R. Copying referral letters to patients: The views of patients, patient representatives and doctors. Patient Educ Couns. 2004;55(1):94–8.

13. Minhas R. Does copying clinical or sharing correspondence to patients result in better care? Vol. 61, International Journal of Clinical Practice. 2007. p. 1390–5.

14. O'Driscoll BR, Koch J, Paschalides C. Copying letters to patients: most patients want copies of letters from outpatient clinics and find them useful. Vol. 327, BMJ (Clinical research ed.). 2003. p. 451.

For peer review only

For peer review only - http://bmjopen.bmj.com/site/about/guidelines.xhtml

15. VanSuch M, Naessens JM, Stroebel RJ, Huddleston JM, Williams AR. Effect of discharge instructions on readmission of hospitalised patients with heart failure: Do all of the Joint Commission on Accreditation of Healthcare Organizations heart failure core measures reflect better care? Qual Saf Heal Care. 2006;15(6):414–7.

16. Kelley K, Clark B, Brown V, Sitzia J. Recruitment process and description of the sample having access to the questionnaire. [cited 2019 Sep 25]; Available from: https://watermark.silverchair.com/mzg031.pdf?token=AQECAHi208BE49Ooan9kkhW_Ercy7Dm3ZL_9Cf3qfKAc485ysgAAAmAwggJcBgkqhkiG9w0BBwagggJNMIICSQIBADCCAkIGCSqGSIb3DQEHATAeBglghkgBZQMEAS4wEQQMvJGQAvtFfKg1-e_MAgEQgIICE6gwobbp5J3ssq2wiiOTQIciDtT5-Ep5xxNYnBo9koEgaxdb

17. Kuranstaltengesetz Kundmachungsorgan K. Krankenanstalten- u. Kuranstaltengesetz § 24 [Internet]. Vol. 124. 2009 [cited 2018 Jan 30]. Available from: www.ris.bka.gv.at

18. Sørensen K, Van Den Broucke S, Fullam J, Doyle G, Pelikan J. Health literacy and public health: A systematic review and integration of definitions and models. BMC Public Health [Internet]. 2012 [cited 2018 Mar 26];12:80. Available from: https://bmcpublichealth.biomedcentral.com/track/pdf/10.1186/1471-2458-12-80?site=bmcpublichealth.biomedcentral.com

19. Bench SD, Heelas K, White C, Griffiths P. Providing critical care patients with a personalised discharge summary: A questionnaire survey and retrospective analysis exploring feasibility and effectiveness. Intensive Crit Care Nurs. 2014;30(2):69–76.

20. Buurman BM, Verhaegh KJ, Smeulers M, Vermeulen H, Geerlings SE, Smorenburg S, et al. Improving handoff communication from hospital to home: the development, implementation

For peer review only

For peer review only - http://bmjopen.bmj.com/site/about/guidelines.xhtml

and evaluation of a personalized patient discharge letter. Int J Qual Heal Care [Internet]. 2016 Jun [cited 2018 Jun 7];28(3):384–90. Available from: http://www.ncbi.nlm.nih.gov/pubmed/27224995

21. Sorita A, Robelia PM, Kattel SB, McCoy CP, Keller AS, Almasri J, et al. The Ideal Hospital Discharge Summary. J Patient Saf. 2017;1.

22. Shilo L, Shilo G. Analysis of abbreviations used by residents in admission notes and discharge summaries. QJM. 2018;111(3):179–83.

23. Shivji FS, Ramoutar DN, Bailey C, Hunter JB. Improving communication with primary care to ensure patient safety post-hospital discharge. Br J Hosp Med. 2015;76(1):46–9.

For peer review only

For peer review only - http://bmjopen.bmj.com/site/about/guidelines.xhtml

TABLES

TABLE 1: Content of the survey

Item	*Questions*
2	**The DS is a communication tool …**
2.1	... among physicians.
2.2	… for the information of patients.
2.3	... for all persons authorized by the patient (legal persons, relatives, caregivers).
2.4	The DS that is comprehensible for patients leads to less time-consuming questions.
2.5	The DS is usually not read by patients.
2.6	The patient needn´t understand the DS, as it is explained by the further treating physicians.
2.7	As part of my education I have attended relevant training courses in the field of communication, such as dealing with patients and relatives.
2.8	In my education as a physician, the structure and content of the DS was an integral part.
2.9	I regularly attend training courses on communication.
3	**The following are necessary contents in the DS …**
3.1	... diagnosis
3.2	... therapy
3.3	… medical terminology
3.4	... specific abbreviations
3.5	... recommendations on further treatment
3.6	... prescription of medication
3.7	... control visits and follow-up appointments
3.8	... behavioral recommendations for the patients
3.9	…therapy recommendations / report from graduated health and nursing staff
3.10	…therapy recommendations / report from the clinical social workers
3.11	…therapy recommendations / report from the medical-technical services (e.g. dietology, physiotherapy)
3.12	The content of the DS in its current form is sufficient for further treatment.
3.13	The DS in its present form contributes to increase the individual health literacy of patients.
4.1	In future, the DS should contain further procedures and treatment goals in an understandable way, so that patients themselves can contribute to the improvement of their health.
4.2	Should the DS be changed in its current form?

For peer review only

For peer review only - http://bmjopen.bmj.com/site/about/guidelines.xhtml

TABLE 2: Baseline characteristics of the participating physicians, presented separately for internal and external physicians

Variable	*Category*	*Internal (n=747)*	*External (n=313)*	*Total (n=1060)*
Gender	Female	346 (46.3%)	114 (36.4%)	460 (43.4%)
	Male	371 (49.7%)	194 (62%)	565 (53.3%)
	(Missing)	30 (4%)	5 (1.6%)	35 (3.3%)
Work experience in years	0-10	276 (36.9%)	33 (10.5%)	309 (29.2%)
	11-20	201 (26.9%)	73 (23.3%)	274 (25.8%)
	>20	258 (34.5%)	203 (64.9%)	461 (43.5%)
	(Missing)	12 (1.6%)	4 (1.3%)	16 (1.5%)
Level of work experience	Junior physicians	37 (5%)	-	37 (3.5%)
	Ward physicians	163 (21.8%)	-	163 (15.4%)
	Assistant physicians	349 (46.7%)	-	349 (32.9%)
	Senior physicians	176 (23.6%)	-	176 (16.6%)
	(Missing)	22 (2.9%)	313 (100%)	335 (31.6%)
Field of work	General practitioner	-	138 (44.1%)	138 (13%)
	Specialist	-	152 (48.6%)	152 (14.3%)
	(Missing)	747 (100%)	23 (7.3%)	770 (72.6%)

Percentages pertain to available responses.

For peer review only

TABLE 3. Responses to the survey items, presented separately for internal and external physicians

For peer review only

For peer review only - http://bmjopen.bmj.com/site/about/guidelines.xhtml

Item	*Category*	*Internal*	*External*	*Total*	*p-value*
2. **The DS is a communication tool …**					
2.1 ... among physicians.	1/2	728 (98.1%)	303 (97.4%)	1031 (97.9%)	0.483
	3/4	14 (1.9%)	8 (2.6%)	22 (2.1%)	
2.2 … for the information of patients.	1/2	559 (75.8%)	208 (67.8%)	767 (73.5%)	0.009*
	3/4	178 (24.2%)	99 (32.2%)	277 (26.5%)	
2.3 ... for all persons authorized by the patient (legal persons, relatives, caregivers).	1/2	478 (66.5%)	186 (61.4%)	664 (65%)	0.132
	3/4	241 (33.5%)	117 (38.6%)	358 (35%)	
2.4 The DS that is comprehensible for patients leads to less time-consuming questions.	1/2	467 (65.8%)	224 (72.7%)	691 (67.9%)	0.034*
	3/4	243 (34.2%)	84 (27.3%)	327 (32.1%)	
2.5 The DS is usually not read by patients.	1/2	315 (47.2%)	145 (48.3%)	460 (47.5%)	0.781
	3/4	353 (52.8%)	155 (51.7%)	508 (52.5%)	
2.6 The patient needn´t understand the DS, as it is explained by the further treating physicians.	1/2	173 (23.9%)	100 (32.3%)	273 (26.4%)	0.006*
	3/4	551 (76.1%)	210 (67.7%)	761 (73.6%)	
2.7 As part of my education I have attended relevant training courses in the field of communication, such as dealing with patients and relatives.	1/2	347 (48.1%)	147 (49.7%)	494 (48.5%)	0.679
	3/4	375 (51.9%)	149 (50.3%)	524 (51.5%)	
2.8 In my education as a physician, the structure and content of the DS was an integral part	1/2	341 (46.8%)	171 (55.5%)	512 (49.4%)	0.012*
	3/4	388 (53.2%)	137 (44.5%)	525 (50.6%)	
2.9 I regularly attend training courses on communication.	1/2	227 (31%)	106 (34.6%)	333 (32.1%)	0.274
	3/4	506 (69%)	200 (65.4%)	706 (67.9%)	
3. **The following are necessary contents in the DS …**					
3.1 ... diagnosis	1/2	733 (100%)	311 (100%)	1044 (100%)	1.000
	3/4	0 (0%)	0 (0%)	0 (0%)	
3.2 ... therapy	1/2	730 (99.6%)	310 (100%)	1040 (99.7%)	0.559
	3/4	3 (0.4%)	0 (0%)	3 (0.3%)	
3.3 … medical terminology	1/2	493 (68.7%)	228 (74.5%)	721 (70.4%)	0.062
	3/4	225 (31.3%)	78 (25.5%)	303 (29.6%)	
3.4 ... specific abbreviations	1/2	162 (22.8%)	65 (21.7%)	227 (22.5%)	0.742
	3/4	547 (77.2%)	235 (78.3%)	782 (77.5%)	
3.5 ... recommendations on further treatment	1/2	729 (99.6%)	308 (99.7%)	1037 (99.6%)	1.000
	3/4	3 (0.4%)	1 (0.3%)	4 (0.4%)	
3.6 ... prescription of medication	1/2	720 (98.5%)	303 (98.4%)	1023 (98.5%)	1.000
	3/4	11 (1.5%)	5 (1.6%)	16 (1.5%)	
3.7 ... control visits and follow-up appointments	1/2	721 (99%)	303 (97.7%)	1024 (98.7%)	0.137
	3/4	7 (1%)	7 (2.3%)	14 (1.3%)	
3.8 ... behavioral recommendations for the patients	1/2	686 (95.3%)	282 (92.5%)	968 (94.4%)	0.075
	3/4	34 (4.7%)	23 (7.5%)	57 (5.6%)	
3.9 …therapy recommendations / report from graduated health and nursing staff	1/2	326 (46.7%)	160 (52.8%)	486 (48.6%)	0.085

For peer review only - http://bmjopen.bmj.com/site/about/guidelines.xhtml

	3/4	372 (53.3%)	143 (47.2%)	515 (51.4%)	
3.10 …therapy recommendations / report from the clinical social workers	1/2	375 (53.6%)	171 (56.8%)	546 (54.5%)	0.368
	3/4	325 (46.4%)	130 (43.2%)	455 (45.5%)	
3.11 …therapy recommendations / report from the medical-technical services (e.g. dietology, physiotherapy)	1/2	473 (67.3%)	207 (69.2%)	680 (67.9%)	0.555
	3/4	230 (32.7%)	92 (30.8%)	322 (32.1%)	
3.12 The content of the DS in its current form is sufficient for further treatment.	1/2	571 (81%)	216 (71.5%)	787 (78.2%)	0.001*
	3/4	134 (19%)	86 (28.5%)	220 (21.8%)	
3.13 The DS in its present form contributes to increase the individual health literacy of patients.	1/2	346 (52.8%)	115 (41.2%)	461 (49.4%)	0.001*
	3/4	309 (47.2%)	164 (58.8%)	473 (50.6%)	
4.1. In future, the DS should contain further procedures and treatment goals in an understandable way, so that patients themselves can contribute to the improvement of their health.	1/2	573 (79.6%)	240 (78.7%)	813 (79.3%)	0.737
	3/4	147 (20.4%)	65 (21.3%)	212 (20.7%)	
4.2 Should the DS be changed in its current form?	1/2	284 (41.4%)	165 (57.9%)	449 (46.2%)	<0.001*
	3/4	402 (58.6%)	120 (42.1%)	522 (53.8%)	

Legend: * significant values, missing data can occur because of nonresponse and is not explicitly stated; percentages pertain to available responses.

For peer review only

For peer review only - http://bmjopen.bmj.com/site/about/guidelines.xhtml

TABLE 4. Responses to the survey items according to gender

Item	*Category*	*Female*	*Male*	*Total*	*p-value*
2. **The DS is a communication tool …**					
2.1 ... among physicians.	1/2	451 (98%)	548 (97.7%)	1031 (97.9%)	0.829
	3/4	9 (2%)	13 (2.3%)	22 (2.1%)	
2.2 … for the information of patients.	1/2	374 (82.2%)	373 (67%)	767 (73.5%)	<0.001*
	3/4	81 (17.8%)	184 (33%)	277 (26.5%)	
2.3 ... for all persons authorized by the patient (legal persons, relatives, caregivers).	1/2	309 (70.4%)	336 (60.9%)	664 (65%)	0.002*
	3/4	130 (29.6%)	216 (39.1%)	358 (35%)	
2.4 The DS that is comprehensible for patients leads to less time-consuming questions.	1/2	321 (71.7%)	353 (65%)	691 (67.9%)	0.029*
	3/4	127 (28.3%)	190 (35%)	327 (32.1%)	
2.5 The DS is usually not read by patients.	1/2	194 (46.3%)	254 (48.8%)	460 (47.5%)	0.470
	3/4	225 (53.7%)	267 (51.2%)	508 (52.5%)	
2.6 The patient needn´t understand the DS, as it is explained by the further treating physicians.	1/2	86 (18.9%)	174 (31.8%)	273 (26.4%)	<0.001*
	3/4	368 (81.1%)	374 (68.2%)	761 (73.6%)	
2.7 As part of my education I have attended relevant training courses in the field of communication, such as dealing with patients and relatives.	1/2	207 (46.8%)	273 (50.1%)	494 (48.5%)	0.337
	3/4	235 (53.2%)	272 (49.9%)	524 (51.5%)	
2.8 In my education as a physician, the structure and content of the DS was an integral part	1/2	209 (46.4%)	286 (51.5%)	512 (49.4%)	0.113
	3/4	241 (53.6%)	269 (48.5%)	525 (50.6%)	
2.9 I regularly attend training courses on communication.	1/2	142 (31.1%)	179 (32.5%)	333 (32.1%)	0.684
	3/4	314 (68.9%)	372 (67.5%)	706 (67.9%)	
3. **The following are necessary contents in the DS …**					
3.1 ... diagnosis	1/2	455 (100%)	557 (100%)	1044 (100%)	1.000
	3/4	0 (0%)	0 (0%)	0 (0%)	
3.2 ... therapy	1/2	453 (99.8%)	555 (99.6%)	1040 (99.7%)	1.000
	3/4	1 (0.2%)	2 (0.4%)	3 (0.3%)	
3.3 … medical terminology	1/2	310 (69.5%)	387 (70.7%)	721 (70.4%)	0.676
	3/4	136 (30.5%)	160 (29.3%)	303 (29.6%)	
3.4 ... specific abbreviations	1/2	76 (17.2%)	140 (26%)	227 (22.5%)	0.001*
	3/4	365 (82.8%)	398 (74%)	782 (77.5%)	
3.5 ... recommendations on further treatment	1/2	450 (99.6%)	555 (99.6%)	1037 (99.6%)	1.000
	3/4	2 (0.4%)	2 (0.4%)	4 (0.4%)	

For peer review only

3.6 ... prescription of medication	1/2	448 (98.7%)	543 (98.2%)	1023 (98.5%)	0.619
	3/4	6 (1.3%)	10 (1.8%)	16 (1.5%)	
3.7 ... control visits and follow-up appointments	1/2	447 (98.7%)	547 (98.7%)	1024 (98.7%)	1.000
	3/4	6 (1.3%)	7 (1.3%)	14 (1.3%)	
3.8 ... behavioral recommendations for the patients	1/2	420 (94.2%)	517 (94.5%)	968 (94.4%)	0.890
	3/4	26 (5.8%)	30 (5.5%)	57 (5.6%)	
3.9 …therapy recommendations / report from graduated health and nursing staff	1/2	218 (50.5%)	252 (46.7%)	486 (48.6%)	0.246
	3/4	214 (49.5%)	288 (53.3%)	515 (51.4%)	
3.10 …therapy recommendations / report from the clinical social workers	1/2	260 (59.5%)	274 (51.1%)	546 (54.5%)	0.010*
	3/4	177 (40.5%)	262 (48.9%)	455 (45.5%)	
3.11 …therapy recommendations / report from the medical-technical services (e.g. dietology, physiotherapy)	1/2	314 (72%)	349 (65%)	680 (67.9%)	0.022*
	3/4	122 (28%)	188 (35%)	322 (32.1%)	
3.12 The content of the DS in its current form is sufficient for further treatment.	1/2	333 (76.2%)	431 (80%)	787 (78.2%)	0.161
	3/4	104 (23.8%)	108 (20%)	220 (21.8%)	
3.13 The DS in its present form contributes to increase the individual health literacy of patients.	1/2	192 (47.9%)	252 (49.9%)	461 (49.4%)	0.548
	3/4	209 (52.1%)	253 (50.1%)	473 (50.6%)	
4.1. In future, the DS should contain further procedures and treatment goals in an understandable way, so that patients themselves can contribute to the improvement of their health.	1/2	364 (81.6%)	426 (77.9%)	813 (79.3%)	0.155
	3/4	82 (18.4%)	121 (22.1%)	212 (20.7%)	
4.2 Should the DS be changed in its current form?	1/2	198 (46%)	238 (46.3%)	449 (46.2%)	0.948
	3/4	232 (54%)	276 (53.7%)	522 (53.8%)	

Legend: * significant values, missing data can occur because of nonresponse and is not explicitly stated; percentages pertain to available responses.

For peer review only

TABLE 5. Responses to the survey items according to years of work experience

Item	*Category*	*0-10*	*11-20*	*>20*	*Total*	*p-value*
**2. The DS is a communication tool …**						
2.1 ... among physicians.	1/2	303 (98.4%)	261 (96%)	454 (98.7%)	1031 (97.9%)	0.050[d]
	3/4	5 (1.6%)	11 (4%)	6 (1.3%)	22 (2.1%)	
2.2 … for the information of patients.	1/2	248 (80.5%)	214 (79.3%)	293 (64.8%)	767 (73.5%)	<0.001[b,c]
	3/4	60 (19.5%)	56 (20.7%)	159 (35.2%)	277 (26.5%)	
2.3 ... for all persons authorized by the patient (legal persons, relatives, caregivers).	1/2	201 (68.4%)	188 (69.4%)	264 (59.5%)	664 (65%)	0.008 [b,c]
	3/4	93 (31.6%)	83 (30.6%)	180 (40.5%)	358 (35%)	
2.4 The DS that is comprehensible for patients leads to less time-consuming questions.	1/2	207 (69.2%)	193 (73.1%)	281 (63.6%)	691 (67.9%)	0.026[c]
	3/4	92 (30.8%)	71 (26.9%)	161 (36.4%)	327 (32.1%)	
2.5 The DS is usually not read by patients.	1/2	157 (56.3%)	119 (47.6%)	178 (41.9%)	460 (47.5%)	0.001[b]
	3/4	122 (43.7%)	131 (52.4%)	247 (58.1%)	508 (52.5%)	
2.6 The patient needn´t understand the DS, as it is explained by the further treating physicians.	1/2	51 (16.7%)	62 (23.5%)	156 (34.6%)	273 (26.4%)	<0.001[b,c]
	3/4	254 (83.3%)	202 (76.5%)	295 (65.4%)	761 (73.6%)	
2.7 As part of my education I have attended relevant training courses in the field of communication, such as dealing with patients and relatives.	1/2	151 (50.3%)	115 (43.7%)	222 (50.1%)	494 (48.5%)	0.196
	3/4	149 (49.7%)	148 (56.3%)	221 (49.9%)	524 (51.5%)	
2.8 In my education as a physician, the structure and content of the DS was an integral part	1/2	128 (42.5%)	137 (50.7%)	241 (53.2%)	512 (49.4%)	0.014[b]
	3/4	173 (57.5%)	133 (49.3%)	212 (46.8%)	525 (50.6%)	
2.9 I regularly attend training courses on communication.	1/2	74 (24.3%)	88 (32.8%)	167 (36.9%)	333 (32.1%)	0.001[b]
	3/4	230 (75.7%)	180 (67.2%)	286 (63.1%)	706 (67.9%)	
**3. The following are necessary contents in the DS …**						
3.1 ... diagnosis	1/2	304 (100%)	273 (100%)	454 (100%)	1044 (100%)	1.000
	3/4	0 (0%)	0 (0%)	0 (0%)	0 (0%)	
3.2 ... therapy	1/2	303 (99.7%)	271 (100%)	453 (99.6%)	1040 (99.7%)	0.794
	3/4	1 (0.3%)	0 (0%)	2 (0.4%)	3 (0.3%)	
3.3 … medical terminology	1/2	196 (64.9%)	194 (72.4%)	321 (72.6%)	721 (70.4%)	0.055
	3/4	106 (35.1%)	74 (27.6%)	121 (27.4%)	303 (29.6%)	
3.4 ... specific abbreviations	1/2	58 (19.6%)	64 (24.1%)	103 (23.6%)	227 (22.5%)	0.347
	3/4	238 (80.4%)	202 (75.9%)	334 (76.4%)	782 (77.5%)	
3.5 ... recommendations on further treatment	1/2	303 (99.7%)	269 (99.3%)	453 (99.8%)	1037 (99.6%)	0.696
	3/4	1 (0.3%)	2 (0.7%)	1 (0.2%)	4 (0.4%)	

For peer review only - http://bmjopen.bmj.com/site/about/guidelines.xhtml

3.6 ... prescription of medication	1/2	300 (99%)	266 (98.2%)	444 (98.2%)	1023 (98.5%)	0.655
	3/4	3 (1%)	5 (1.8%)	8 (1.8%)	16 (1.5%)	
3.7 ... control visits and follow-up appointments	1/2	299 (99.7%)	270 (99.3%)	442 (97.6%)	1024 (98.7%)	0.041[d]
	3/4	1 (0.3%)	2 (0.7%)	11 (2.4%)	14 (1.3%)	
3.8 ... behavioral recommendations for the patients	1/2	290 (97.3%)	254 (94.1%)	411 (92.6%)	968 (94.4%)	0.016[b,c]
	3/4	8 (2.7%)	16 (5.9%)	33 (7.4%)	57 (5.6%)	
3.9 …therapy recommendations / report from graduated health and nursing staff	1/2	133 (45.2%)	124 (48.1%)	221 (50.6%)	486 (48.6%)	0.364
	3/4	161 (54.8%)	134 (51.9%)	216 (49.4%)	515 (51.4%)	
3.10 …therapy recommendations / report from the clinical social workers	1/2	166 (56.3%)	134 (51.3%)	239 (55.1%)	546 (54.5%)	0.476
	3/4	129 (43.7%)	127 (48.7%)	195 (44.9%)	455 (45.5%)	
3.11 …therapy recommendations / report from the medical-technical services (e.g. dietology, physiotherapy)	1/2	201 (67.9%)	168 (65.1%)	302 (69.1%)	680 (67.9%)	0.552
	3/4	95 (32.1%)	90 (34.9%)	135 (30.9%)	322 (32.1%)	
3.12 The content of the DS in its current form is sufficient for further treatment.	1/2	234 (79.6%)	203 (78.1%)	340 (77.1%)	787 (78.2%)	0.724
	3/4	60 (20.4%)	57 (21.9%)	101 (22.9%)	220 (21.8%)	
3.13 The DS in its present form contributes to increase the individual health literacy of patients.	1/2	145 (52.3%)	129 (52.7%)	182 (45.3%)	461 (49.4%)	0.095
	3/4	132 (47.7%)	116 (47.3%)	220 (54.7%)	473 (50.6%)	
4.1. In future, the DS should contain further procedures and treatment goals in an understandable way, so that patients themselves can contribute to the improvement of their health.	1/2	245 (81.1%)	224 (84.2%)	333 (75.2%)	813 (79.3%)	0.011[c]
	3/4	57 (18.9%)	42 (15.8%)	110 (24.8%)	212 (20.7%)	
4.2 Should the DS be changed in its current form?	1/2	114 (39.3%)	121 (49%)	209 (49.6%)	449 (46.2%)	0.016 [b]
	3/4	176 (60.7%)	126 (51%)	212 (50.4%)	522 (53.8%)	

409 Legend: Missing data can occur because of nonresponse and is not explicitly stated; percentages pertain to available responses.

410 [a] Significant differences (p<0.017) between 0-10 and 11-20 years

411 [b] Significant differences (p<0.017) between 0-10 and >20 years

412 [c] Significant differences (p<0.017) between 11-20 and >20 years

413 [d] No pairwise difference remained significant after Bonferroni correction

For peer review only - http://bmjopen.bmj.com/site/about/guidelines.xhtml

Declarations

Contributors:

Gurantor: Magdalena Hoffmann

Study concept and design: Magdalena Hoffmann, Gernot Brunner, Maximilian Weinrauch, Gerald Sendlhofer

Acquisition of data: Magdalena Hoffmann, Christine Maria Schwarz, Lydia Jantscher, Lars-Peter Kamolz

Analysis and interpretation of pooled data: Magdalena Hoffmann, Christine Maria Schwarz, Gudrun Pregartner, Lydia Jantscher, Lars-Peter Kamolz

Drafting of the manuscript: Magdalena Hoffmann, Christine Maria Schwarz, Gerald Sendlhofer

Critical revision of the manuscript for important intellectual content: All authors

Statistical analysis of pooled data: Gudrun Pregartner, Lydia Jantscher

Funding: This work was supported by the Healthcare fund Styria (Gesundheitsfonds Steiermark). The funders had no role in the study design, data collection, data analysis, data interpretation, or writing of the report.

Competing interests: All authors have completed the ICMJE uniform disclosure form at http://icmje.org/conflicts-of-interest/and declared no conflict of interests.

Patient consent for publication: Not required.

Ethics approval: The Ethics Committee of the Medical University of Graz approved the study (vote#: 30-220 ex17/18).

For peer review only

434 **Transparency declaration:** The lead author (MH) affirms that this manuscript is an honest,
435 accurate, and transparent account of the study being reported; that no important aspects of the study
436 have been omitted; and that any discrepancies from the study as planned have been explained.

437 **Consent for publication**: All authors confirmed the publication in its present form.

438 **Data availability statement:** The datasets used and/or analyzed during the current study are
439 available from the corresponding author on reasonable request.

For peer review only

For peer review only - http://bmjopen.bmj.com/site/about/guidelines.xhtml

Supplemental Table 1

External General practitioner vs specialist

variable	category	1	2	Total	p (Chi-sq.)	p (Fisher)
**2.1**	1/2	134 (97.1%)	148 (97.4%)	282 (97.2%)	0.890	1.000
	3/4	4 (2.9%)	4 (2.6%)	8 (2.8%)		
**2.2**	1/2	94 (69.1%)	97 (65.1%)	191 (67%)	0.471	0.529
	3/4	42 (30.9%)	52 (34.9%)	94 (33%)		
**2.3**	1/2	81 (60.9%)	89 (59.3%)	170 (60.1%)	0.788	0.809
	3/4	52 (39.1%)	61 (40.7%)	113 (39.9%)		
**2.4**	1/2	104 (76.5%)	101 (67.3%)	205 (71.7%)	0.087	0.090
	3/4	32 (23.5%)	49 (32.7%)	81 (28.3%)		
**2.5**	1/2	62 (46.6%)	73 (49.7%)	135 (48.2%)	0.611	0.633
	3/4	71 (53.4%)	74 (50.3%)	145 (51.8%)		
**2.6**	1/2	46 (33.6%)	49 (32.5%)	95 (33%)	0.839	0.900
	3/4	91 (66.4%)	102 (67.5%)	193 (67%)		
**2.7**	1/2	68 (50.7%)	73 (51%)	141 (50.9%)	0.960	1.000
	3/4	66 (49.3%)	70 (49%)	136 (49.1%)		
**2.8**	1/2	70 (51.5%)	91 (60.3%)	161 (56.1%)	0.134	0.153
	3/4	66 (48.5%)	60 (39.7%)	126 (43.9%)		
**2.9**	1/2	50 (36.2%)	49 (33.1%)	99 (34.6%)	0.579	0.620
	3/4	88 (63.8%)	99 (66.9%)	187 (65.4%)		
**3.1**	1/2	137 (100%)	152 (100%)	289 (100%)	-	-
	3/4	0 (0%)	0 (0%)	0 (0%)		
**3.2**	1/2	137 (100%)	151 (100%)	288 (100%)	-	-
	3/4	0 (0%)	0 (0%)	0 (0%)		
**3.3**	1/2	102 (75%)	110 (73.8%)	212 (74.4%)	0.821	0.892
	3/4	34 (25%)	39 (26.2%)	73 (25.6%)		
**3.4**	1/2	22 (16.5%)	36 (24.7%)	58 (20.8%)	0.095	0.106
	3/4	111 (83.5%)	110 (75.3%)	221 (79.2%)		
**3.5**	1/2	135 (99.3%)	151 (100%)	286 (99.7%)	0.291	0.474
	3/4	1 (0.7%)	0 (0%)	1 (0.3%)		
**3.6**	1/2	135 (99.3%)	147 (98%)	282 (98.6%)	0.363	0.624
	3/4	1 (0.7%)	3 (2%)	4 (1.4%)		
**3.7**	1/2	135 (99.3%)	147 (96.7%)	282 (97.9%)	0.130	0.218
	3/4	1 (0.7%)	5 (3.3%)	6 (2.1%)		
**3.8**	1/2	125 (91.9%)	136 (91.9%)	261 (91.9%)	0.995	1.000
	3/4	11 (8.1%)	12 (8.1%)	23 (8.1%)		
**3.9**	1/2	84 (61.8%)	68 (46.6%)	152 (53.9%)	0.011	0.012
	3/4	52 (38.2%)	78 (53.4%)	130 (46.1%)		
**3.10**	1/2	89 (65.4%)	72 (50%)	161 (57.5%)	0.009	0.011
	3/4	47 (34.6%)	72 (50%)	119 (42.5%)		
**3.11**	1/2	98 (73.1%)	98 (67.6%)	196 (70.3%)	0.311	0.359
	3/4	36 (26.9%)	47 (32.4%)	83 (29.7%)		
**3.12**	1/2	93 (68.9%)	106 (72.6%)	199 (70.8%)	0.494	0.514
	3/4	42 (31.1%)	40 (27.4%)	82 (29.2%)		

variable	category	1	2	Total	p (Chi-sq.)	p (Fisher)
**3.13**	1/2	60 (46.5%)	45 (34.6%)	105 (40.5%)	0.051	0.058
	3/4	69 (53.5%)	85 (65.4%)	154 (59.5%)		
**4.1**	1/2	109 (80.1%)	114 (77%)	223 (78.5%)	0.522	0.565
	3/4	27 (19.9%)	34 (23%)	61 (21.5%)		
**4.2**	1/2	84 (65.1%)	70 (51.1%)	154 (57.9%)	0.021	0.025
	3/4	45 (34.9%)	67 (48.9%)	112 (42.1%)		

For peer review only

Fisher- vs. Chi-Quadrat-Test

Physicians Supplemental Table 2

variable	category	Internal	External	Total	p (Chi-sq)	p (Fisher)
**2.1**	1/2	728 (98.1%)	303 (97.4%)	1031 (97.9%)	0.478	0.483
	3/4	14 (1.9%)	8 (2.6%)	22 (2.1%)		
**2.2**	1/2	559 (75.8%)	208 (67.8%)	767 (73.5%)	0.007	0.009
	3/4	178 (24.2%)	99 (32.2%)	277 (26.5%)		
**2.3**	1/2	478 (66.5%)	186 (61.4%)	664 (65%)	0.119	0.132
	3/4	241 (33.5%)	117 (38.6%)	358 (35%)		
**2.4**	1/2	467 (65.8%)	224 (72.7%)	691 (67.9%)	0.029	0.034
	3/4	243 (34.2%)	84 (27.3%)	327 (32.1%)		
**2.5**	1/2	315 (47.2%)	145 (48.3%)	460 (47.5%)	0.734	0.781
	3/4	353 (52.8%)	155 (51.7%)	508 (52.5%)		
**2.6**	1/2	173 (23.9%)	100 (32.3%)	273 (26.4%)	0.005	0.006
	3/4	551 (76.1%)	210 (67.7%)	761 (73.6%)		
**2.7**	1/2	347 (48.1%)	147 (49.7%)	494 (48.5%)	0.643	0.679
	3/4	375 (51.9%)	149 (50.3%)	524 (51.5%)		
**2.8**	1/2	341 (46.8%)	171 (55.5%)	512 (49.4%)	0.010	0.012
	3/4	388 (53.2%)	137 (44.5%)	525 (50.6%)		
**2.9**	1/2	227 (31%)	106 (34.6%)	333 (32.1%)	0.248	0.274
	3/4	506 (69%)	200 (65.4%)	706 (67.9%)		
**3.1**	1/2	733 (100%)	311 (100%)	1044 (100%)	-	-
	3/4	0 (0%)	0 (0%)	0 (0%)		
**3.2**	1/2	730 (99.6%)	310 (100%)	1040 (99.7%)	0.259	0.559
	3/4	3 (0.4%)	0 (0%)	3 (0.3%)		
**3.3**	1/2	493 (68.7%)	228 (74.5%)	721 (70.4%)	0.061	0.062
	3/4	225 (31.3%)	78 (25.5%)	303 (29.6%)		
**3.4**	1/2	162 (22.8%)	65 (21.7%)	227 (22.5%)	0.681	0.742
	3/4	547 (77.2%)	235 (78.3%)	782 (77.5%)		
**3.5**	1/2	729 (99.6%)	308 (99.7%)	1037 (99.6%)	0.837	1.000
	3/4	3 (0.4%)	1 (0.3%)	4 (0.4%)		
**3.6**	1/2	720 (98.5%)	303 (98.4%)	1023 (98.5%)	0.887	1.000
	3/4	11 (1.5%)	5 (1.6%)	16 (1.5%)		
**3.7**	1/2	721 (99%)	303 (97.7%)	1024 (98.7%)	0.097	0.137
	3/4	7 (1%)	7 (2.3%)	14 (1.3%)		
**3.8**	1/2	686 (95.3%)	282 (92.5%)	968 (94.4%)	0.072	0.075
	3/4	34 (4.7%)	23 (7.5%)	57 (5.6%)		
**3.9**	1/2	326 (46.7%)	160 (52.8%)	486 (48.6%)	0.076	0.085
	3/4	372 (53.3%)	143 (47.2%)	515 (51.4%)		
**3.10**	1/2	375 (53.6%)	171 (56.8%)	546 (54.5%)	0.345	0.368
	3/4	325 (46.4%)	130 (43.2%)	455 (45.5%)		
**3.11**	1/2	473 (67.3%)	207 (69.2%)	680 (67.9%)	0.546	0.555
	3/4	230 (32.7%)	92 (30.8%)	322 (32.1%)		
**3.12**	1/2	571 (81%)	216 (71.5%)	787 (78.2%)	0.001	0.001

variable	category	Internal	External	Total	p (Chi-sq)	p (Fisher)
	3/4	134 (19%)	86 (28.5%)	220 (21.8%)		
**3.13**	1/2	346 (52.8%)	115 (41.2%)	461 (49.4%)	0.001	0.001
	3/4	309 (47.2%)	164 (58.8%)	473 (50.6%)		
**4.1**	1/2	573 (79.6%)	240 (78.7%)	813 (79.3%)	0.746	0.737
	3/4	147 (20.4%)	65 (21.3%)	212 (20.7%)		
**4.2**	1/2	284 (41.4%)	165 (57.9%)	449 (46.2%)	<0.001	<0.001
	3/4	402 (58.6%)	120 (42.1%)	522 (53.8%)		

For peer review only

International Journal for Quality in Health Care 2003; **Volume 15, Number 3**: pp. 261–266
10.1093/intqhc/mzg031

Methodology Matters

Good practice in the conduct and reporting of survey research

KATE KELLEY, BELINDA CLARK, VIVIENNE BROWN AND JOHN SITZIA

Research Department, Worthing & Southlands Hospitals NHS Trust, Worthing, West Sussex, UK

Abstract

Survey research is sometimes regarded as an easy research approach. However, as with any other research approach and method, it is easy to conduct a survey of poor quality rather than one of high quality and real value. This paper provides a checklist of good practice in the conduct and reporting of survey research. Its purpose is to assist the novice researcher to produce survey work to a high standard, meaning a standard at which the results will be regarded as credible. The paper first provides an overview of the approach and then guides the reader step-by-step through the processes of data collection, data analysis, and reporting. It is not intended to provide a manual of how to conduct a survey, but rather to identify common pitfalls and oversights to be avoided by researchers if their work is to be valid and credible.

Keywords: data reporting, health care surveys, methodology, questionnaires, research design, survey methods, surveys

For peer review only

What is survey research?

Survey research is common in studies of health and health services, although its roots lie in the social surveys conducted in Victorian Britain by social reformers to collect information on poverty and working class life (e.g. Charles Booth [1] and Joseph Rowntree [2]), and indeed survey research remains most used in applied social research. The term 'survey' is used in a variety of ways, but generally refers to the selection of a relatively large sample of people from a pre-determined population (the 'population of interest'; this is the wider group of people in whom the researcher is interested in a particular study), followed by the collection of a relatively small amount of data from those individuals. The researcher therefore uses information from a sample of individuals to make some inference about the wider population.

Data are collected in a standardized form. This is usually, but not necessarily, done by means of a questionnaire or interview. Surveys are designed to provide a 'snapshot of how things are at a specific time' [3]. There is no attempt to control conditions or manipulate variables; surveys do not allocate participants into groups or vary the treatment they receive. Surveys are well suited to descriptive studies, but can also be used to explore aspects of a situation, or to seek explanation and provide data for testing hypotheses. It is important to recognize that 'the survey approach is a research strategy, not a research method' [3]. As with any research approach, a choice of methods is available and the one most appropriate to the individual project should be used. This paper will discuss the most popular methods employed in survey research, with an emphasis upon difficulties commonly encountered when using these methods.

Descriptive research

Descriptive research is a most basic type of enquiry that aims to observe (gather information on) certain phenomena, typically at a single point in time: the 'cross-sectional' survey. The aim is to examine a situation by describing important factors associated with that situation, such as demographic, socio-economic, and health characteristics, events, behaviours, attitudes, experiences, and knowledge. Descriptive studies are used to estimate specific parameters in a population (e.g. the prevalence of infant breast feeding) and to describe associations (e.g. the association between infant breast feeding and maternal age).

Analytical studies

Analytical studies go beyond simple description; their intention is to illuminate a specific problem through focused

Address reprint requests to John Sitzia, Research Department, Worthing Hospital, Lyndhurst Road, Worthing BN11 2DH, West Sussex, UK. E-mail: john.sitzia@wash.nhs.uk

© International Society for Quality in Health Care and Oxford University Press 2003; all rights reserved

For peer review only - http://bmjopen.bmj.com/site/about/guidelines.xhtml

data analysis, typically by looking at the effect of one set of variables upon another set. These are longitudinal studies, in which data are collected at more than one point in time with the aim of illuminating the direction of observed associations. Data may be collected from the same sample on each occasion (cohort or panel studies) or from a different sample at each point in time (trend studies).

Evaluation research

This form of research collects data to ascertain the effects of a planned change.

Advantages and disadvantages of survey research

Advantages:

- The research produces data based on real-world observations (empirical data).
- The breadth of coverage of many people or events means that it is more likely than some other approaches to obtain data based on a representative sample, and can therefore be generalizable to a population.
- Surveys can produce a large amount of data in a short time for a fairly low cost. Researchers can therefore set a finite time-span for a project, which can assist in planning and delivering end results.

Disadvantages:

- The significance of the data can become neglected if the researcher focuses too much on the range of coverage to the exclusion of an adequate account of the implications of those data for relevant issues, problems, or theories.
- The data that are produced are likely to lack details or depth on the topic being investigated.
- Securing a high response rate to a survey can be hard to control, particularly when it is carried out by post, but is also difficult when the survey is carried out face-to-face or over the telephone.

Essential steps in survey research

Research question

Good research has the characteristic that its purpose is to address a single clear and explicit research question; conversely, the end product of a study that aims to answer a number of diverse questions is often weak. Weakest of all, however, are those studies that have no research question at all and whose design simply is to collect a wide range of data and then to 'trawl' the data looking for 'interesting' or 'significant' associations. This is a trap novice researchers in particular fall into. Therefore, in developing a research question, the following aspects should be considered [4]:

- Be knowledgeable about the area you wish to research.
- Widen the base of your experience, explore related areas, and talk to other researchers and practitioners in the field you are surveying.
- Consider using techniques for enhancing creativity, for example brainstorming ideas.
- Avoid the pitfalls of: allowing a decision regarding methods to decide the questions to be asked; posing research questions that cannot be answered; asking questions that have already been answered satisfactorily.

Research methods

The survey approach can employ a range of methods to answer the research question. Common survey methods include postal questionnaires, face-to-face interviews, and telephone interviews.

Postal questionnaires

This method involves sending questionnaires to a large sample of people covering a wide geographical area. Postal questionnaires are usually received 'cold', without any previous contact between researcher and respondent. The response rate for this type of method is usually low, ~20%, depending on the content and length of the questionnaire. As response rates are low, a large sample is required when using postal questionnaires, for two main reasons: first, to ensure that the demographic profile of survey respondents reflects that of the survey population; and secondly, to provide a sufficiently large data set for analysis.

Face-to-face interviews

Face-to-face interviews involve the researcher approaching respondents personally, either in the street or by calling at people's homes. The researcher then asks the respondent a series of questions and notes their responses. The response rate is often higher than that of postal questionnaires as the researcher has the opportunity to sell the research to a potential respondent. Face-to-face interviewing is a more costly and time-consuming method than the postal survey, however the researcher can select the sample of respondents in order to balance the demographic profile of the sample.

Telephone interviews

Telephone surveys, like face-to-face interviews, allow a two-way interaction between researcher and respondent. Telephone surveys are quicker and cheaper than face-to-face interviewing. Whilst resulting in a higher response rate than postal surveys, telephone surveys often attract a higher level of refusals than face-to-face interviews as people feel less inhibited about refusing to take part when approached over the telephone.

Designing the research tool

Whether using a postal questionnaire or interview method, the questions asked have to be carefully planned and piloted.

For peer review only - http://bmjopen.bmj.com/site/about/guidelines.xhtml

The design, wording, form, and order of questions can affect the type of responses obtained, and careful design is needed to minimize bias in results. When designing a questionnaire or question route for interviewing, the following issues should be considered: (1) planning the content of a research tool; (2) questionnaire layout; (3) interview questions; (4) piloting; and (5) covering letter.

Planning the content of a research tool

The topics of interest should be carefully planned and relate clearly to the research question. It is often useful to involve experts in the field, colleagues, and members of the target population in question design in order to ensure the validity of the coverage of questions included in the tool (content validity).

Researchers should conduct a literature search to identify existing, psychometrically tested questionnaires. A well designed research tool is simple, appropriate for the intended use, acceptable to respondents, and should include a clear and interpretable scoring system. A research tool must also demonstrate the psychometric properties of reliability (consistency from one measurement to the next), validity (accurate measurement of the concept), and, if a longitudinal study, responsiveness to change [5]. The development of research tools, such as attitude scales, is a lengthy and costly process. It is important that researchers recognize that the development of the research tool is equal in importance—and deserves equal attention—to data collection. If a research instrument has not undergone a robust process of development and testing, the credibility of the research findings themselves may legitimately be called into question and may even be completely disregarded. Surveys of patient satisfaction and similar are commonly weak in this respect; one review found that only 6% of patient satisfaction studies used an instrument that had undergone even rudimentary testing [6]. Researchers who are unable or unwilling to undertake this process are strongly advised to consider adopting an existing, robust research tool.

Questionnaire layout

Questionnaires used in survey research should be clear and well presented. The use of capital (upper case) letters only should be avoided, as this format is hard to read. Questions should be numbered and clearly grouped by subject. Clear instructions should be given and headings included to make the questionnaire easier to follow.

The researcher must think about the form of the questions, avoiding 'double-barrelled' questions (two or more questions in one, e.g. 'How satisfied were you with your personal nurse and the nurses in general?'), questions containing double negatives, and leading or ambiguous questions. Questions may be open (where the respondent composes the reply) or closed (where pre-coded response options are available, e.g. multiple-choice questions). Closed questions with pre-coded response options are most suitable for topics where the possible responses are known. Closed questions are quick to administer and can be easily coded and analysed. Open questions should be used where possible replies are unknown or too numerous to pre-code. Open questions are more demanding for respondents but if well answered can provide useful insight into a topic. Open questions, however, can be time consuming to administer and difficult to analyse. Whether using open or closed questions, researchers should plan clearly how answers will be analysed.

Interview questions

Open questions are used more frequently in unstructured interviews, whereas closed questions typically appear in structured interview schedules. A structured interview is like a questionnaire that is administered face to face with the respondent. When designing the questions for a structured interview, the researcher should consider the points highlighted above regarding questionnaires. The interviewer should have a standardized list of questions, each respondent being asked the same questions in the same order. If closed questions are used the interviewer should also have a range of pre-coded responses available.

If carrying out a semi-structured interview, the researcher should have a clear, well thought out set of questions; however, the questions may take an open form and the researcher may vary the order in which topics are considered.

Piloting

A research tool should be tested on a pilot sample of members of the target population. This process will allow the researcher to identify whether respondents understand the questions and instructions, and whether the meaning of questions is the same for all respondents. Where closed questions are used, piloting will highlight whether sufficient response categories are available, and whether any questions are systematically missed by respondents.

When conducting a pilot, the same procedure as as that to be used in the main survey should be followed; this will highlight potential problems such as poor response.

Covering letter

All participants should be given a covering letter including information such as the organization behind the study, including the contact name and address of the researcher, details of how and why the respondent was selected, the aims of the study, any potential benefits or harm resulting from the study, and what will happen to the information provided. The covering letter should both encourage the respondent to participate in the study and also meet the requirements of informed consent (see below).

Sample and sampling

The concept of sample is intrinsic to survey research. Usually, it is impractical and uneconomical to collect data from every single person in a given population; a sample of the population has to be selected [7]. This is illustrated in the following

For peer review only - http://bmjopen.bmj.com/site/about/guidelines.xhtml

hypothetical example. A hospital wants to conduct a satisfaction survey of the 1000 patients discharged in the previous month; however, as it is too costly to survey each patient, a sample has to be selected. In this example, the researcher will have a list of the population members to be surveyed (sampling frame). It is important to ensure that this list is both up-to date and has been obtained from a reliable source.

The method by which the sample is selected from a sampling frame is integral to the external validity of a survey: the sample has to be representative of the larger population to obtain a composite profile of that population [8].

There are methodological factors to consider when deciding who will be in a sample: How will the sample be selected? What is the optimal sample size to minimize sampling error? How can response rates be maximized?

The survey methods discussed below influence how a sample is selected and the size of the sample. There are two categories of sampling: random and non-random sampling, with a number of sampling selection techniques contained within the two categories. The principal techniques are described here [9].

Random sampling

Generally, random sampling is employed when quantitative methods are used to collect data (e.g. questionnaires). Random sampling allows the results to be generalized to the larger population and statistical analysis performed if appropriate. The most stringent technique is simple random sampling. Using this technique, each individual within the chosen population is selected by chance and is equally as likely to be picked as anyone else. Referring back to the hypothetical example, each patient is given a serial identifier and then an appropriate number of the 1000 population members are randomly selected. This is best done using a random number table, which can be generated using computer software (a free on-line randomizer can be found at http://www.randomizer.org/index.htm).

Alternative random sampling techniques are briefly described. In systematic sampling, individuals to be included in the sample are chosen at equal intervals from the population; using the earlier example, every fifth patient discharged from hospital would be included in the survey. Stratified sampling selects a specific group and then a random sample is selected. Using our example, the hospital may decide only to survey older surgical patients. Bigger surveys may employ cluster sampling, which randomly assigns groups from a large population and then surveys everyone within the groups, a technique often used in national-scale studies.

Non-random sampling

Non-random sampling is commonly applied when qualitative methods (e.g. focus groups and interviews) are used to collect data, and is typically used for exploratory work. Non-random sampling deliberately targets individuals within a population. There are three main techniques. (1) purposive sampling: a specific population is identified and only its members are included in the survey; using our example above, the hospital may decide to survey only patients who had an appendectomy. (2) Convenience sampling: the sample is made up of the individuals who are the easiest to recruit. Finally, (3) snowballing: the sample is identified as the survey progresses; as one individual is surveyed he or she is invited to recommend others to be surveyed.

It is important to use the right method of sampling and to be aware of the limitations and statistical implications of each. The need to ensure that the sample is representative of the larger population was highlighted earlier and, alongside the sampling method, the degree of sampling error should be considered. Sampling error is the probability that any one sample is not completely representative of the population from which it has been drawn [9]. Although sampling error cannot be eliminated entirely, the sampling technique chosen will influence the extent of the error. Simple random sampling will give a closer estimate of the population than a convenience sample of individuals who just happened to be in the right place at the right time.

Sample size

What sample size is required for a survey? There is no definitive answer to this question: large samples with rigorous selection are more powerful as they will yield more accurate results, but data collection and analysis will be proportionately more time consuming and expensive. Essentially, the target sample size for a survey depends on three main factors: the resources available, the aim of the study, and the statistical quality needed for the survey. For 'qualitative' surveys using focus groups or interviews, the sample size needed will be smaller than if quantitative data is collected by questionnaire. If statistical analysis is to be performed on the data then sample size calculations should be conducted. This can be done using computer packages such as G*Power [10]; however, those with little statistical knowledge should consult a statistician. For practical recommendations on sample size, the set of survey guidelines developed by the UK Department of Health [11] should be consulted.

Larger samples give a better estimate of the population but it can be difficult to obtain an adequate number of responses. It is rare that everyone asked to participate in the survey will reply. To ensure a sufficient number of responses, include an estimated non-response rate in the sample size calculations.

Response rates are a potential source of bias. The results from a survey with a large non-response rate could be misleading and only representative of those who replied. French [12] reported that non-responders to patient satisfaction surveys are less likely to be satisfied than people who reply. It is unwise to define a level above which a response rate is acceptable, as this depends on many local factors; however, an achievable and acceptable rate is ~75% for interviews and 65% for self-completion postal questionnaires [9,13]. In any study, the final response rate should be reported with the results; potential differences between the respondents and non-respondents should be explicitly explored and their implications discussed.

For peer review only - http://bmjopen.bmj.com/site/about/guidelines.xhtml

There are techniques to increase response rates. A questionnaire must be concise and easy to understand, reminders should be sent out, and method of recruitment should be carefully considered. Sitzia and Wood [13] found that participants recruited by mail or who had to respond by mail had a lower mean response rate (67%) than participants who were recruited personally (mean response 76.7%). A most useful review of methods to maximize response rates in postal surveys has recently been published [14].

Data collection

Researchers should approach data collection in a rigorous and ethical manner. The following information must be clearly recorded:

- How, where, how many times, and by whom potential respondents were contacted.
- How many people were approached and how many of those agreed to participate.
- How did those who agreed to participate differ from those who refused with regard to characteristics of interest in the study, for example how were they identified, where were they approached, and what was their gender, age, and features of their illness or health care.
- How was the survey administered (e.g. telephone interview).
- What was the response rate (i.e. the number of usable data sets as a proportion of the number of people approached).

Data analysis

The purpose of all analyses is to summarize data so that it is easily understood and provides the answers to our original questions: 'In order to do this researchers must carefully examine their data; they should become friends with their data' [15]. Researchers must prepare to spend substantial time on the data analysis phase of a survey (and this should be built into the project plan). When analysis is rushed, often important aspects of the data are missed and sometimes the wrong analyses are conducted, leading to both inaccurate results and misleading conclusions [16]. However, and this point cannot be stressed strongly enough, researchers must not engage in data dredging, a practice that can arise especially in studies in which large numbers of dependent variables can be related to large numbers of independent variables (outcomes). When large numbers of possible associations in a dataset are reviewed at $P < 0.05$, one in 20 of the associations by chance will appear 'statistically significant'; in datasets where only a few real associations exist, testing at this significance level will result in the large majority of findings still being false positives [17].

The method of data analysis will depend on the design of the survey and should have been carefully considered in the planning stages of the survey. Data collected by qualitative methods should be analysed using established methods such as content analysis [18], and where quantitative methods have been used appropriate statistical tests can be applied. Describing methods of analysis here would be unproductive as a multitude of introductory textbooks and on-line resources are available to help with simple analyses of data (e.g. [19, 20]). For advanced analysis a statistician should be consulted.

Reporting

When reporting survey research, it is essential that a number of key points are covered (though the length and depth of reporting will be dependent upon journal style). These key points are presented as a 'checklist' below:

(1) Explain the purpose or aim of the research, with the explicit identification of the research question.
(2) Explain why the research was necessary and place the study in context, drawing upon previous work in relevant fields (the literature review).
(3) Describe in (proportionate) detail how the research was done.
  (a) State the chosen research method or methods, and justify why this method was chosen.
  (b) Describe the research tool. If an existing tool is used, briefly state its psychometric properties and provide references to the original development work. If a new tool is used, you should include an entire section describing the steps undertaken to develop and test the tool, including results of psychometric testing.
  (c) Describe how the sample was selected and how data were collected, including:
    (i) How were potential subjects identified?
    (ii) How many and what type of attempts were made to contact subjects?
    (iii) Who approached potential subjects?
    (iv) Where were potential subjects approached?
    (v) How was informed consent obtained?
    (vi) How many agreed to participate?
    (vii) How did those who agreed differ from those who did not agree?
    (viii) What was the response rate?
(4) Describe and justify the methods and tests used for data analysis.
(5) Present the results of the research. The results section should be clear, factual, and concise.
(6) Interpret and discuss the findings. This 'discussion' section should not simply reiterate results; it should provide the author's critical reflection upon both the results and the processes of data collection. The discussion should assess how well the study met the research question, should describe the problems encountered in the research, and should honestly judge the limitations of the work.
(7) Present conclusions and recommendations.

The researcher needs to tailor the research report to meet:

For peer review only - http://bmjopen.bmj.com/site/about/guidelines.xhtml

- The expectations of the specific audience for whom the work is being written.
- The conventions that operate at a general level with respect to the production of reports on research in the social sciences.

Ethics

Anyone involved in collecting data from patients has an ethical duty to respect each individual participant's autonomy. Any survey should be conducted in an ethical manner and one that accords with best research practice. Two important ethical issues to adhere to when conducting a survey are confidentiality and informed consent.

The respondent's right to confidentiality should always be respected and any legal requirements on data protection adhered to. In the majority of surveys, the patient should be fully informed about the aims of the survey, and the patient's consent to participate in the survey must be obtained and recorded.

The professional bodies listed below, among many others, provide guidance on the ethical conduct of research and surveys.

- American Psychological Association: http://www.apa.org
- British Psychological Society: http://www.bps.org.uk
- British Medical Association: http://www.bma.org.uk.
- UK General Medical Council: http://www.gmc-uk.org
- American Medical Association: http://www.ama-assn.org
- UK Royal College of Nursing: http://www.rcn.org.uk
- UK Department of Health: http://www.doh.gov

Conclusion

Survey research demands the same standards in research practice as any other research approach, and journal editors and the broader research community will judge a report of survey research with the same level of rigour as any other research report. This is not to say that survey research need be particularly difficult or complex; the point to emphasize is that researchers should be aware of the steps required in survey research, and should be systematic and thoughtful in the planning, execution, and reporting of the project. Above all, survey research should not be seen as an easy, 'quick and dirty' option; such work may adequately fulfil local needs (e.g. a quick survey of hospital staff satisfaction), but will not stand up to academic scrutiny and will not be regarded as having much value as a contribution to knowledge.

References

1. London School of Economics, UK. Http://booth.lse.ac.uk/ (accessed 15 January 2003).
2. Vernon A. *A Quaker Businessman: Biography of Joseph Rowntree (1836–1925)*. London: Allen & Unwin, 1958.
3. Denscombe M. *The Good Research Guide: For Small-scale Social Research Projects*. Buckingham: Open University Press, 1998.
4. Robson C. *Real World Research: A Resource for Social Scientists and Practitioner-researchers*. Oxford: Blackwell Publishers, 1993.
5. Streiner DL, Norman GR. *Health Measurement Scales: A Practical Guide to their Development and Use*. Oxford: Oxford University Press, 1995.
6. Sitzia J. How valid and reliable are patient satisfaction data? An analysis of 195 studies. *Int J Qual Health Care* 1999; **11:** 319–328.
7. Bowling A. *Research Methods in Health. Investigating Health and Health Services*. Buckingham: Open University Press, 2002.
8. American Statistical Association, USA. Http://www.amstat.org (accessed 9 December 2002).
9. Arber S. Designing samples. In: Gilbert N, ed. *Researching Social Life*. London: SAGE Publications, 2001.
10. Heinrich Heine University, Dusseldorf, Germany. Http://www.psycho.uni-duesseldorf.de/aap/projects/gpower/index.html (accessed 12 December 2002).
11. Department of Health, England. Http://www.doh.gov.uk/acutesurvey/index.htm (accessed 12 December 2002).
12. French K. Methodological considerations in hospital patient opinion surveys. *Int J Nurs Stud* 1981; **18:** 7–32.
13. Sitzia J, Wood N. Response rate in patient satisfaction research: an analysis of 210 published studies. *Int J Qual Health Care* 1998; **10:** 311–317.
14. Edwards P, Roberts I, Clarke M *et al.* Increasing response rates to postal questionnaires: systematic review. *Br Med J* 2002; **324:** 1183.
15. Wright DB. Making friends with our data: improving how statistical results are reported. *Br J Educ Psychol* 2003; in press.
16. Wright DB, Kelley K. Analysing and reporting data. In: Michie S, Abraham C, eds. *Health Psychology in Practice*. London: SAGE Publications, 2003; in press.
17. Davey Smith G, Ebrahim S. Data dredging, bias, or confounding. *Br Med J* 2002; **325:** 1437–1438.
18. Morse JM, Field PA. *Nursing Research: The Application of Qualitative Approaches*. London: Chapman and Hall, 1996.
19. Wright DB. *Understanding Statistics: An Introduction for the Social Sciences*. London: SAGE Publications, 1997.
20. Sportscience, New Zealand. Http://www.sportsci.org/resource/stats/index.html (accessed 12 December 2002).

Accepted for publication 16 January 2003

For peer review only

For peer review only - http://bmjopen.bmj.com/site/about/guidelines.xhtml
